# Intraocular nano-microscale drug delivery systems for glaucoma treatment: design strategies and recent progress

**DOI:** 10.1186/s12951-023-01838-x

**Published:** 2023-03-10

**Authors:** Yuening Shen, Jianguo Sun, Xinghuai Sun

**Affiliations:** 1 Department of Ophthalmology & Visual Science, Eye & ENT Hospital, Shanghai Medical College, Fudan University, 83 Fenyang Road, Xuhui District, Shanghai, 200031 China; 2 State Key Laboratory of Medical Neurobiology and MOE Frontiers Center for Brain Science, Institutes of Brain Science, Fudan University, Shanghai, 200032 China; 3 NHC Key Laboratory of Myopia, Chinese Academy of Medical Sciences, and Shanghai Key Laboratory of Visual Impairment and Restoration (Fudan University), Shanghai, 200031 China

**Keywords:** Glaucoma, Nanomedicine, Drug delivery, Sustained release, Neuroprotection, Intraocular pressure

## Abstract

Glaucoma is a leading cause of irreversible visual impairment and blindness, affecting over 76.0 million people worldwide in 2020, with a predicted increase to 111.8 million by 2040. Hypotensive eye drops remain the gold standard for glaucoma treatment, while inadequate patient adherence to medication regimens and poor bioavailability of drugs to target tissues are major obstacles to effective treatment outcomes. Nano/micro-pharmaceuticals, with diverse spectra and abilities, may represent a hope of removing these obstacles. This review describes a set of intraocular nano/micro drug delivery systems involved in glaucoma treatment. Particularly, it investigates the structures, properties, and preclinical evidence supporting the use of these systems in glaucoma, followed by discussing the route of administration, the design of systems, and factors affecting in vivo performance. Finally, it concludes by highlighting the emerging notion as an attractive approach to address the unmet needs for managing glaucoma.

## Introduction

### Glaucoma

Glaucoma is a leading cause of irreversible visual impairment and blindness worldwide [1–3]. Individuals with glaucoma were estimated to be 76.0–79.6 million in 2020 and this number may rise to over 111.8 million by 2040 [3, 4]. The global glaucoma prevalence in the population at the age of 40–80 was calculated to be approximately 3.54% [4, 5]. Glaucoma is known as a “silent thief of vision” because warning signs are usually subtle and symptoms only felt in the late stages when the visual field has already been compromised severely [5–7].

**Fig. 1:**
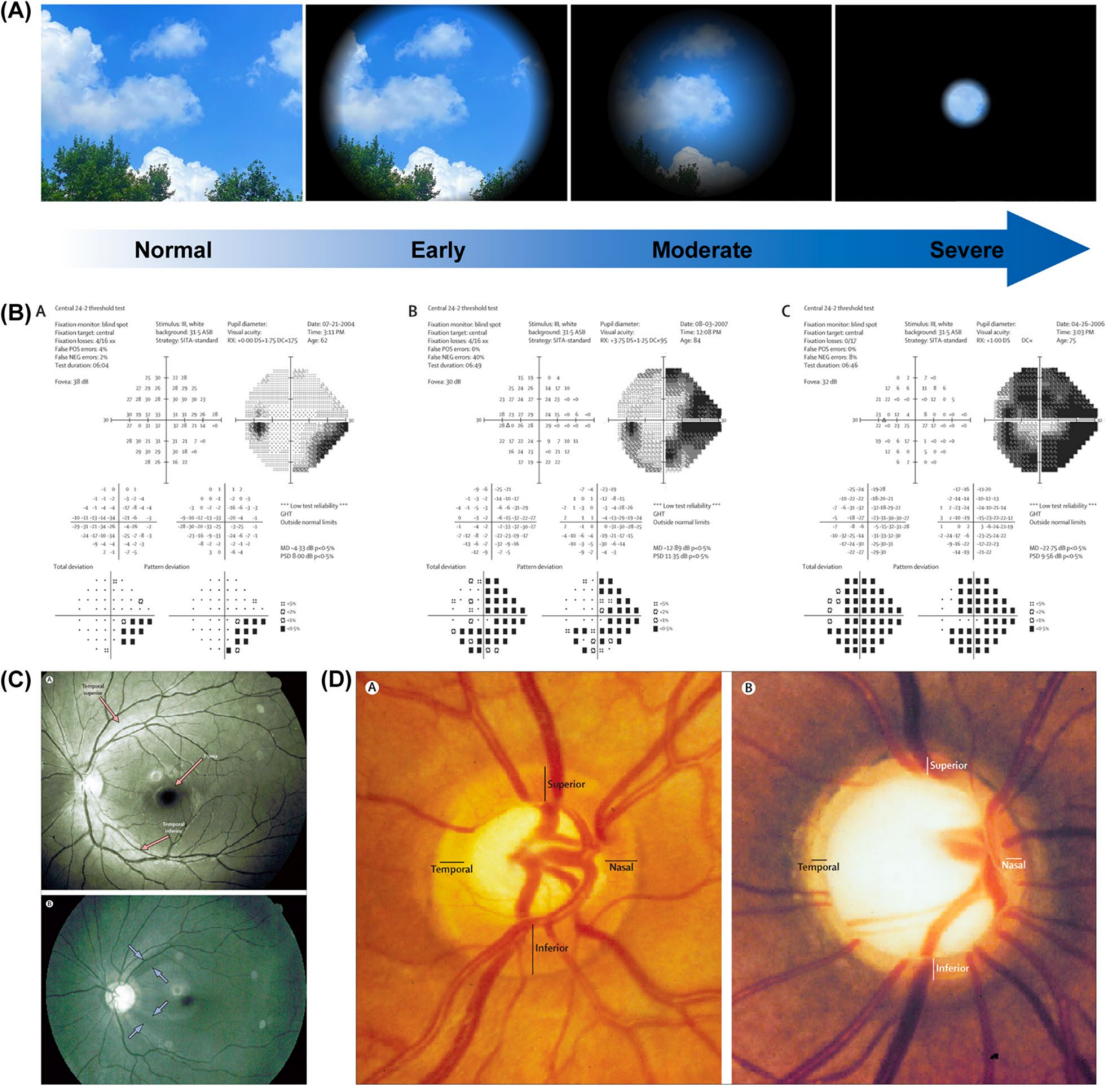
Progressive defect of the visual field and the loss of retinal nerve fibre layer in glaucoma. **A** Normal vision and vision in glaucoma patients. Patients usually experience blurry or missing spots in peripheral vision at early-stage disease. At nearly end-stage disease, only a central vision remains and “tunnel vision” is generated. **B** Visual field tests of glaucomatous left eyes show early (**A**), moderate (**B**), and severe (**C**) stages of functional loss. Reprinted from Ref. [6] with permission from Elsevier. **C** The ophthalmoscopic photograph of the retinal nerve fibre layer in healthy individuals (**A**) show a healthy retinal nerve fibre layer without any defect (red arrows). In patients with glaucoma (**B**), there are localised reduced reflexes of the retinal nerve fibre layer (light blue arrows), indicating the diminution of retinal nerve fibre layer. Reprinted from Ref. [5] with permission from Elsevier. **D** The optic disc of healthy individuals without glaucoma shows a normal shape of the neuroretinal rim, with its widest part in the inferior region (**A**). With glaucoma damage, the cup becomes deeper and larger, and the rim is much thinner than in the healthy optic disc (**B**). Reprinted from Ref. [5] with permission from Elsevier

Glaucoma has been recognized as a multifactorial neurodegenerative disorder and its pathogenesis remains not fully elucidated [8, 9]. It is a group of diseases characterized by structural damage and loss of retinal nerve fibre layer (retinal ganglion cells (RGCs)) in pathology, and progressive defect of the visual field in clinical manifestation [5, 6, 10] (Fig. 1). Although many risk factors have been identified, such as ocular structural predisposition, increased intraocular pressure (IOP) is the only modifiable risk factor at present [11–13]. IOP is most often controlled by the daily dose of IOP-lowering eye drops [11, 14]. Current anti-glaucoma management is conducted in a stepwise fashion and starts with single topical hypotensive eye drops [11]. These eye drop medications typically lower the IOP through alteration of aqueous humour dynamics, either reducing its production (beta-blockers, alpha-agonists, carbonic anhydrase inhibitors) or increasing its outflow (pilocarpine, epinephrine, prostaglandin analogues (PGA)) [12, 13]. If initial monotherapies are not sufficient to control the IOP, multi-drug treatments, laser and/or surgical interventions are employed [11, 12]. On the other hand, the concept of “neuroprotection” (i.e. treatments independent of IOP reduction intending to prevent or delay RGCs and axonal death) has also received increasing attention, since the disruption of functional connectivity in the optic nerve is indicated in glaucoma pathophysiology [15–17]. Glaucomatous RGC damage is a multifactorial neurodegenerative process, whose possible mechanisms include but are not limited to the aggregation of misfolded proteins, neuroinflammation, oxidative stress, mitochondrial dysfunction, and neurotrophin support reduction [18–21]. Simple reduction and maintenance of IOP may not be sufficient to prevent the progressive loss of the visual field [9, 10, 22–24]. Neuroprotective strategies have shown promising treatment outcomes in animal models, many of which are under clinical trials, but none of them has been applied in clinical practice to date [22].

### Issues with current treatment regimens

Topical administration of IOP-lowering eye drops is a relatively non-invasive and simple route for drug delivery, which is the current gold standard for treatment [25, 26]. However, the efficacy of treatment is undermined by patients’ inadequate adherence to medication regimens and limited bioavailability of drugs to target sites [9, 12].

An ideal medication instillation requires the right timing, frequency, dose, and better accompanied with skills to prolong the preservation time on the eye surface (e.g. pressing the dacryocyst area after the instillation) [27–29]. However, objective studies have demonstrated poor patient adherence on average. In some cases, more than half of patients have deviated from their prescribed medication regimens [9, 30–39]. Common barriers to medication adherence include low self-efficacy, forgetfulness, and difficulties with eye drop administration [35]. Taking medications that require more than twice per day, taking adjunctive treatments, or undergoing changes of medications also seem to be the factors decreasing patient adherence [40–43]. Patient compliance may be optimized when applying monotherapy or electronic monitoring [9, 27, 44], but neither of them is feasible for each patient in a clinical setting at least for now.

Additionally, it is reported that over 60% of patients are struggling with self-administering eye drops [45, 46], and only 39% of patients can complete the instillation properly without touching the ocular surface [28, 45]. These findings have been confirmed by later studies in Asia: less than a half of the patients are able to administrate eye drops on their first attempt; no more than 0.05% of patients are aware of pressing the dacryocyst area after instillations; over 62% of patinets got contact with the ocular surface during the administration [47, 48]. Contact with the ocular surface during instillation contributes to the contamination of eye drop bottles, which is of particular concern in patients who have accepted glaucoma surgeries [13, 49, 50]. It is estimated that 19% of eye drops become contaminated within 8 weeks, and 29–40% for bottles used longer [49, 50].

It is also found that age-related factors (e.g. ﻿reduced cognition, arthritis, and paralysis) and poor eyesight are responsible for worse self-administration techniques [28, 47, 51], especially in identifying medications, squeezing drops from bottles, and checking whether drops are delivered [13, 28, 45]. Moreover, the financial burden and adverse effects (AEs) of life-long treatment may add more noncompliance to medical regimens as well [13, 29, 35, 52]. Adherence is critical for the stabilization of the visual field. Studies have shown that patients with 80% adherence are more likely to hinder visual field progression, while those with 21% adherence demonstrate progressive visual field defects [40, 53].

**Fig. 2:**
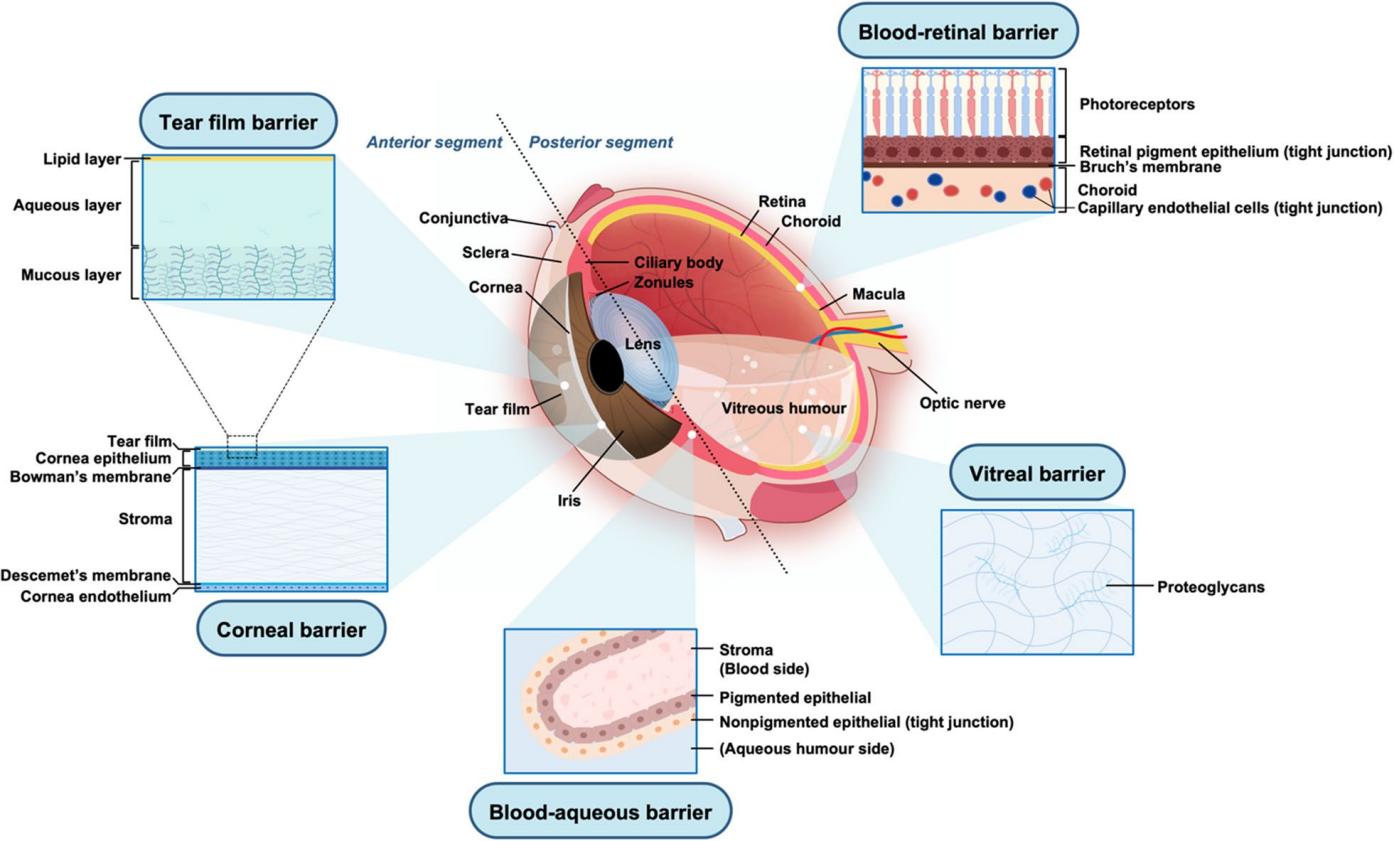
Eye structures and ocular barriers

Bioavailability refers to the extent of drug absorption and is commonly described as the percentage of dose absorption [9]. Delivering drugs to intraocular target tissues through topically administered medications is a long-standing challenge due to the presence of anatomical (statics barriers, such as the cornea, blood-aqueous and blood-retinal barriers) and physiological (dynamic barriers, such as tear drainage, conjunctival blood and lymph flow) barriers of the human eyes [12, 54, 55] (Fig. 2). When medication is given topically as eye drops, anatomical barriers retard drug absorption into intraocular tissues and dynamic barriers rapidly drain the drug into the systemic circulation. Meanwhile, secondary factors, such as blinking, tear film turnover, and nasolacrimal drainage accelerate the elimination of the drug [26, 55]. It is estimated that only 10 μL of the instilled formulation remains on the ocular surface after a single eye blink [56], and almost all drug agents are eliminated from the ocular surface after 15–25 minutes [57, 58]. Eventually, only 5% at best of topically administered drug agents may overcome the hindrance and access the anterior segment, thus frequent administration is required [14, 25, 59–66]. These ocular barriers also contribute to the wax and wane drug effect before and after each administration of the eye drops [9]. Pulsatile drug concentrations may lead to IOP fluctuation at different time points of the day, which is likely to be a risk factor in glaucoma progression [9, 14, 67].

Topical administration of IOP-lowering eye drops certainly remains the cornerstone of anti-glaucoma treatment [25, 26]. However, the aforementioned problems result in poor bioavailability of drugs and non-adherence of patients, which has urged researchers to focus on novel therapeutics with improved treatment efficacy. This need may be met through the employment of nanomedicine [12, 68, 69].

### Nanomedicine

The term “nano” originated from the Greek word meaning “dwarf” [70], and “nanotechnology” is used to describe materials and devices that are measured at a range of 1–100 nm in at least one dimension [70, 71]. Nanomedicine generally refers to the application of nanosystems (< 1000 nm in size) in the diagnosis and treatment of diseases [72–74]. The incorporation of drugs into nanocarriers may surpass the limitations of current treatment regimens by enhancing drug penetration, achieving targeted delivery, prolonging contact of drugs with ocular tissues, and sustaining in vivo release [8, 12, 62, 73, 75, 76]. Not only that, nanocarriers are equally effective in delivering lipophilic drugs, proteins, and even genes, which are difficult with conventional solvents [62, 63]. Nanocarriers are also able to protect the integrity of drug cargo before reaching the target sites. This property is particularly intriguing when transporting molecules such as neurotrophin and antibodies because these proteins easily degrade in vivo [62, 73].

**Fig. 3:**
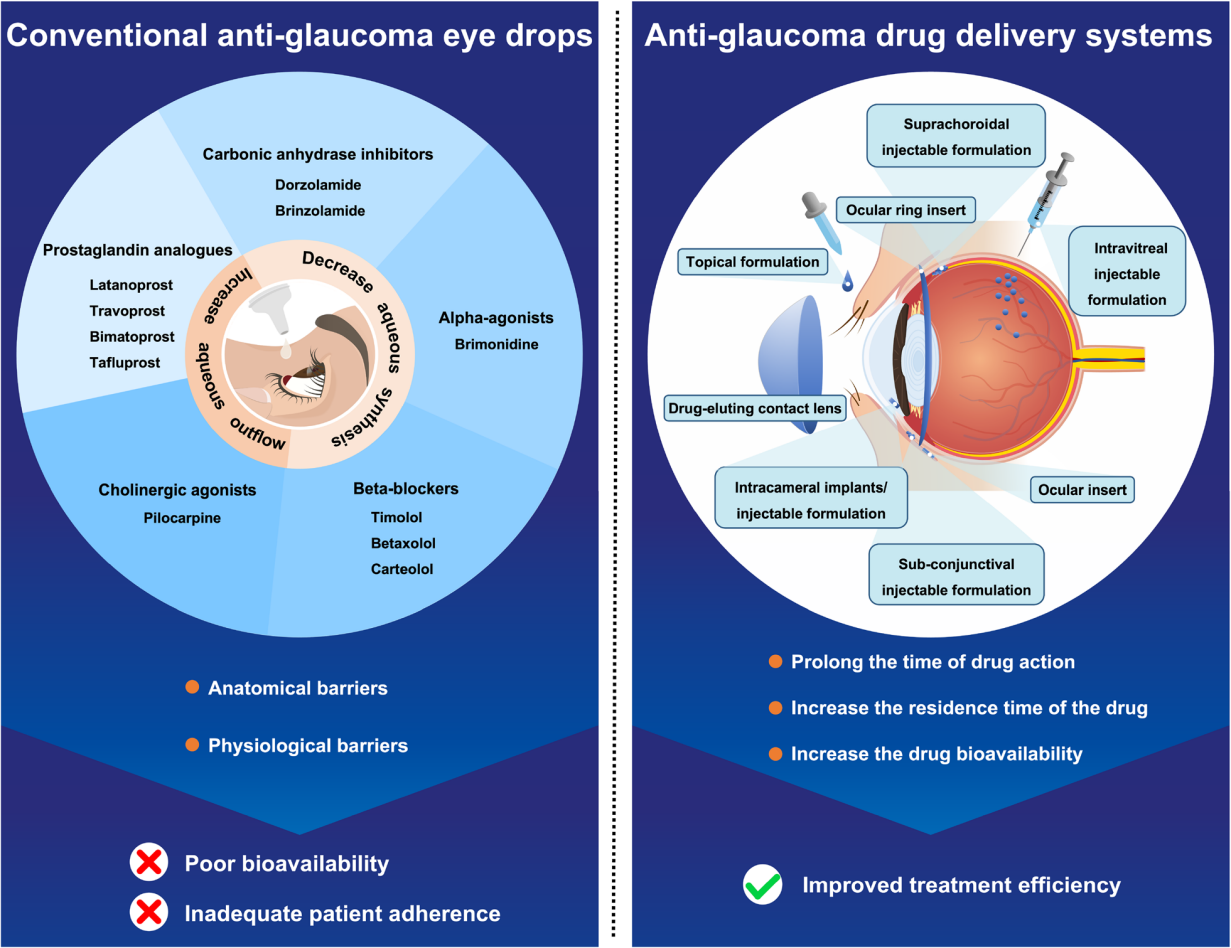
Conventional eye drops and nanotechnology-based therapies for glaucoma treatment

Versatile periocular, extraocular and intraocular nano/micro-drug delivery systems (DDSs) have been engineered, many of which demonstrate satisfactory safety and promising treatment efficacy in glaucoma animal models (Fig. 3). They include topical formulation [77], ocular insert [78], drug-eluting contact lens [79], ocular ring insert [80], intracameral [81], intravitreal [82], subconjunctival [83] and suprachoroidal [84] injectable formulation/implants. Compared with intraocular DDSs, periocular and extraocular DDSs are certainly much less invasive and are easier to administrate or remove [9]. However, ocular barriers are bypassed at most, and bioavailability is maximized for DDSs delivered via intraocular routes [9]. This review will focus on anti-glaucoma intraocular nano-microscale DDSs. Herein, considerations in their design and route for administration are discussed. The commonalities and factors affecting in vivo performance of nanocarriers will then be introduced, followed by a summary of structures, formation, properties, and in vivo pharmacological responses of DDSs. Subsequently, DDSs under clinical trials will be introduced as well. Finally, this review will be concluded by focusing on challenges in clinical translation and future perspectives.

## Design and consideration of drug delivery systems

### General consideration

There are common physicochemical properties that all the nanomaterials selected to form a DDS must possess. They include biocompatibility (absence of toxicity), in vivo stability, and feasibility of sterilization. Furthermore, the carrier should provide superior pharmacological effects than conventional medications (e.g., sustained release, target delivery, enhanced cellular-level penetration).

#### Biocompatibility

Biocompatibility is a key subject to be determined before using a nanomaterial as a drug carrier [12, 25, 85]. ﻿Noxious effects of nanomaterials in living tissues can be induced through various mechanisms, such as the generation of oxidative stress [86] and the disruption of cell membranes [87]. The nanomaterial itself and finally assembled DDSs should be biologically compatible in vivo without triggering cellular toxicity or inflammatory responses [85, 88].

Various in vitro and in vivo assays, including platelet aggregation, macrophages uptake, cell morphology and viability, clinical sign evaluation, gross pathology and histology, have been suggested to evaluate the toxicity of nanomaterials [85, 88]. However, hundreds of transporter types exist on the surface of human cells, and there is also a large difference in the microenvironment between health and illness [8]. Once the materials are administrated into the eyes, it is challenging to identify the exact behaviour and effect. Comprehensive safety studies of nanomaterials remain inadequate [88].

The vital factors affecting the biocompatibility of carrier materials are their physicochemical properties, which mainly include size, surface shape, charge, and chemical groups on the surface [8, 89]. Since mammalian cells are negatively charged, subjects with strong positive charges may destroy the cell membrane [90–93]. Subjects with a smaller size typically exhibit more capabilities in penetrating the cell membrane, consequently leading to more cell or tissue toxicity [94–97]. For instance, silica NPs sized 15 nm display higher retinal cytotoxicity than 50 nm-sized ones in vitro and in vivo [98]. However, properties that may have negative effects in vivo are often what makes these materials attractive as drug carriers [85]. For example, cationic or small-sized carriers with superior abilities of disrupting the cell-lipid bilayer lead to a better interaction between drugs and target tissues at the cellular level [94–97]. Transfection efficiency will be improved when delivering genes [99, 100]. The balance between the desired capabilities and the accompanying potential negative effects should be addressed.

#### Physical stabilization

An ideal nanocarrier should have stable characteristics and not change dramatically after being administrated into living tissues. Take nanoparticles (NPs), the most common form of drug carriers, for examples. Small NPs tend to aggregate in vivo because they are unstable thermodynamically [25, 85]. This aggregation may lead to an extremely high accumulation of drugs at certain sites [85]. NPs also tend to adsorb plasma proteins onto the surface [85]. Hence, caution must be paid when performing an intravitreal injection of NPs, because blood-retina barrier impairment may occur during this procedure. ﻿Currently, transmission electron microscopy (TEM) is the commonly used strategy to observe the distribution and morphology of nanocarriers in living tissues [101]. For fluorescent-labelled nanocarriers, observation with fluorescent microscopes is also a viable alternative [102]. However, the aforementioned methods can only provide a general trend. There is still a huge gap concerning the exact behaviour of nanocarriers in an intraocular environment, especially for degradation and elimination [25, 103].

#### Proper sterilization techniques

Regardless of the forms or the materials used to deliver the drug cargo, the assembled DDSs should be sterile before the final administration. However, proper and convenient sterilization techniques have become a limiting requirement when developing DDSs, as many sterilization methods have been shown to alter the physiochemical properties of carrier materials and drug molecules [25, 104, 105].

Ethylene oxide, gamma irradiation, and autoclaving are the most commonly used sterilization methods for pharmaceutical products and medical devices [106]. During autoclaved sterilization, high temperature and pressure frequently results in physical instability and aggregation of polymers [25, 105]. Gamma irradiation has been proven to be effective with some nanomaterials [107, 108], but free radicals produced in the process can induce structural changes and physical instability [109–111], especially when the loading agent is a protein [112]. Accelerated drug release from its carrier after gamma irradiation was also reported before [108].

Ultraviolet (UV) light and filtration are familiar and economical sterilization methods, but UV light may contribute to increased polymer wettability [113]. Filtration utilizing a 0.20–0.22 µm sterile film may be a practical method to expel contaminants without changing the physicochemical properties of nanomaterials [114, 115]. However, this strategy may not be applicable to NPs with larger sizes as they may experience entrapment inside the membrane [25]. It is also worth mentioning that adding antimicrobial agents to drug carriers can be very risky [25]. DDSs are typically designed to continuously release the loading drugs and remain in the eye for a relatively long time. Long-term application of antimicrobial agents such as benzalkonium chloride is associated with serious side effects [116–118].

Perhaps there is no universal sterilization process suitable for all nanosystems [115]. Utilizing different sterilization techniques for different components separately and completing manufacturing under aseptic conditions may be a practical way [25]. The sterilization strategy should be validated on a case-by-case basis [115].

### Routes of administration

#### Intracameral delivery *versus* intravitreal delivery

A unique advantage of delivering drugs via intraocular routes is that ocular barriers are bypassed and drugs are immediately available at target sites, and consequently, bioavailability is improved [9]. The general approaches to drug delivery via intraocular routes are intracameral and intravitreal injections.

Intracameral injection is applied in present clinical practice for anaesthesia and ocular inflammation [61]. Researchers believe that this route may be suitable for delivering anti-glaucoma drugs as well. Intracameral delivery allows for direct contact between drug agents and anterior segment tissues involved in glaucoma pathology (e.g., the ciliary body, trabecular meshwork and uveoscleral outflow pathways), leading to the rapid increase and high concentration of drugs in the anterior chamber [9]. In this way, drug bioavailability is 100% and a much lower total dose of drugs is required compared with topical medications [9, 13, 61]. However, intracameral delivery is inefficient in delivering drugs to the retina [9]. The posterior segment of the eye is better targeted by the intravitreal route of administration [9, 119]. Intravitreal delivery refers to administrating drug solution/suspension into the vitreous humour via pars plana with a sterile needle. Hence, a higher concentration of drugs in the internal eye and more direct contact of drugs with the retinal ganglion cell layer and the optic nerve head can be achieved in this way [9, 61, 63]. Intravitreal injections may be more acceptable for patients since it has been routinely used for various ocular conditions, such as uveitis, neovascular age-related macular degeneration, and diabetic retinopathy [9, 120]. Certainly, there are complications for both approaches, especially with repeated injections, such as intraocular infection, endophthalmitis, cataract, retinal detachment and haemorrhage, corneal and scleral damage [9, 13, 121, 122]. In the study of intracameral implants using rabbit eyes, partial corneal opacification and neovascularization were observed [123]. Cautions must be paid no matter which route is used for administration.

From the pharmacokinetic point of view, intracamerally administrated drugs are predominantly concentrated in the anterior chamber and difficult to reach the retina [9]. Hence, the intracameral route may be more suitable for IOP-lowering treatments than neuroprotection targeting at the retina. In contrast, intravitreal drugs can be cleared both anteriorly and posteriorly due to their access to the ciliary body, aqueous humour outflow, and the retina [9, 124]. Thus, intravitreal delivery can be a viable route for both IOP reduction and RGCs neuroprotection. Nevertheless, the intravitreal route has not been widely explored in IOP control therapy. On the other hand, intravitreal lipophilic drugs tend to be cleared posteriorly via the retina-choroid circulation, while intravitreal hydrophilic drugs are more likely to be cleared anteriorly via the aqueous humour outflow [125–127]. In other words, the increase of drug lipophilicity reduces the extent of drugs entering into the anterior segment, resulting in a weaker hypotensive effect [9]. Therefore, treatment goal (IOP control or neuroprotection), routes of administration (intracameral delivery or intravitreal delivery), and physicochemical properties of drugs (the extent of lipophilicity and hydrophilicity) should be considered together when designing DDSs.

#### Tolerance of intracameral and intravitreal spaces

Drug-loaded nanocarriers are typically administrated into the eye in the form of suspension or as an implant. The volume of injection or the number and size of the implant should be compatible with the model eyes because the tolerance of external suspension/implants that can be administrated is not infinite. In current studies, the suspension is most used when the DDS is administrated intravitreally. The common solution used for dispersing drug-loaded particles to form a nano-formulation includes a balanced solution and an isotonic phosphate buffer solutions of pH 7.4 [105]. Implants are often seen during the use of intracameral delivery. Implants are generally delivered through an incision near the limbus, and typically, only one implant is administrated per eye.

The amount of suspension/implant required for treatment in vivo depends on the therapeutic window of the drug itself, the drug payload in carriers, and the in vivo release kinetics of the drug cargo from its carriers [105, 128]. The upper limit of the dose is limited by (1) the maximum volume/size that does not trigger a spike in IOP [129], and (2) the tolerance of intraocular concentration of the delivered drug and its products [130]. The former is generally determined by the species of animal models (Table 1); the latter is influenced by the solubility and the intraocular metabolism of the drug delivered, as well as its release pattern from the drug carrier [130, 131].

**Table 1:** Intraocular volumes of different species

Species	﻿Mean iridocorneal angle (degrees ± SD)	Average anterior chamber volume (mL)	Vitreous volume (mL)	References
Rat	40.9 ± 7.6	0.0136	0.013–0.054	[132–134]
Rabbit	31.15 ± 9.30	0.287	1.5–1.8	[130, 132, 135]
Dog	42.4 ± 4	0.770	3.0	[130, 132, 135]
NHP	﻿34 ± 2 (Cynomolgus monkey) 36 ± 1 (Rhesus monkey)	0.123	1.8–2.0	[130, 132, 135]
Human	35.8 ± 12.2	0.310	4.0	[130, 132, 136]

SD, standard deviation; NHP, non-human primates

Rats and rabbits are common model choices for studies on anti-glaucoma intraocular DDSs. ﻿A rat vitreous volume can be considered as approximately 20 µL and an intravitreal injection volume of less than 5 µL is generally considered to have a low risk of AEs [133]. The normal depth of the rabbit anterior chamber is about 2.08 mm [137] and an intracameral injection normally should be within the range of 50 µL [119]. In addition to the volume, the density of materials administrated should also cause no mechanical trauma or severe inflammatory response [130]. In animal studies using rats, intravitreal injection of poly (lactic-co-glycolic acid) (PLGA) microspheres greater than 0.5 mg may induce retinal stress and neuronal cell dysfunctions [138]; 2-µL mix-sized PLGA microspheres of intracameral delivery can form angular aggregation and cause the rise of IOP [139, 140].

Regarding implants, the compatible size and fitness of the implant within the anterior chamber structures are key factors for safety prediction since the implant tends to stay within the confines of the inferior angle after the administration. Otherwise, device migration or restriction, and anterior synechia are likely to happen [123], especially for narrow iridocorneal angles or angles with an anatomical obstruction such as scarring [9, 141, 142].

#### Feasibility of administration

Syringeability and injectability are two key factors that guarantee the administration of the prescribed dose of DDSs with minimal damage to ocular structures [105]. Syringeability means that DDS can pass and be withdrawn by needles, and the finer needles employed, the less invasiveness to the eyes. Injectability refers to the performance of the DDSs during the injection [105]. If clumping occurs, pseudoplastic polymers such as hyaluronic acid can be used to relieve the blockage and improve the syringeability and injectability [105, 143, 144].

Drug carriers with larger sizes typically have higher drug loading capacity and longer drug release duration [145, 146]. For DDSs as a form of suspension, extensive use of large-sized subjects results in poor injectability, such as the clumping of particles in the needle and more backflow from the injection site [145, 146]. For DDSs as an implant, larger-sized implants require greater access with severer invasiveness to complete the administration. In conclusion, a balance should be made between the loading capacity of the drug carrier and the feasibility of administration when designing a DDS.

### Drug carriers

#### Factors affecting in vivo behaviours of drug carriers

Although the specific behaviour of drug carriers in an intraocular environment has not been elucidated, the size and surface charge of particles are believed to determine their intraocular performance [8, 104, 119]. The vitreous humour is an isotonic clear gel-like network mainly consisting of water (98–99%), hyaluronic acid, collagen and proteoglycans [119]. It has a loose structure with an estimated mesh size of 550 nm [147], making it difficult to act as a severe barrier for particle diffusion, but the increase of particle size reduces intravitreal mobility [104, 124, 148, 149]. From a different angle, restricted particles may be seen as a localized system that provides sustained drug delivery to the retina [104]. Small-sized particles typically possess better retinal cell uptake than large-sized particles, but too small particles may be cleared rapidly in vivo, resulting in unsustained drug release [8, 68]. Since the vitreous is negatively charged, cationic particles aggregate with biomacromolecules in the vitreous cavity, leading to restricted diffusion [150–152]; in the contrast, anionic and neutral particles diffuse well and are more likely to penetrate the retina [147, 149, 151]. It is also reported that the shape of particles plays a role in the performance of NPs in vivo. For instance, spherical mesoporous silica nanoparticles (MSNs) are cleared faster than rod ones during renal excretion [103]. Other physicochemical properties, including stiffness [153, 154], hydrophobicity [154] and topography [155], on the surface of nanomaterials have also been found to affect the bio-performance. However, there are few studies on these properties in an intraocular environment.

#### Forms of drug carriers

**Fig. 4:**
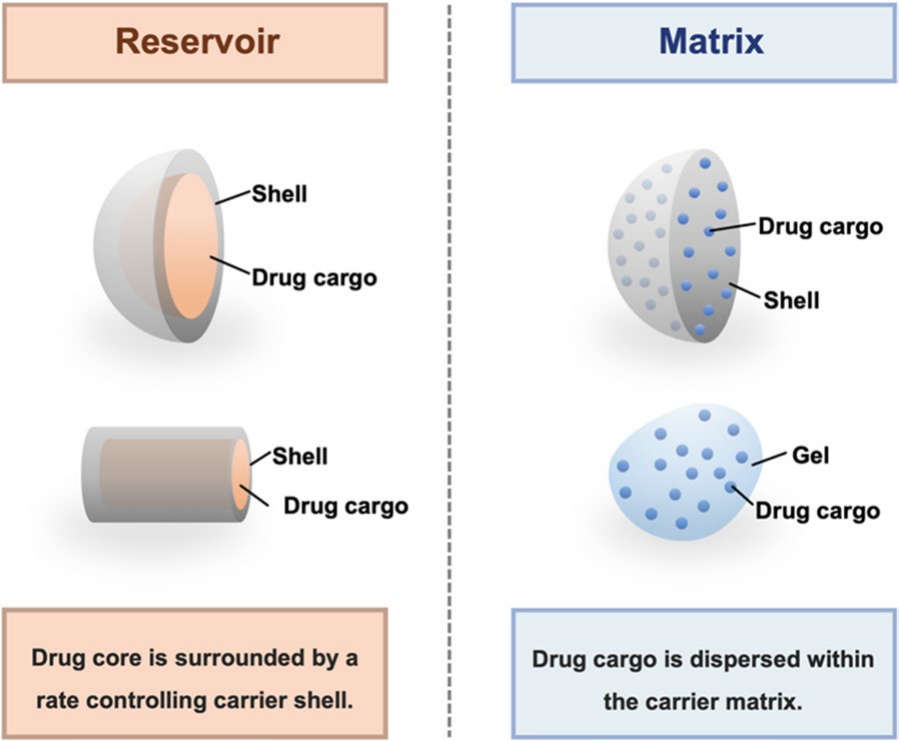
Examples of reservoir-type and matrix-type drug delivery systems

Nanocarriers can be prepared in a variety of forms, such as polymeric nanomicelles (﻿self-assembled suspension of amphiphilic block copolymers with hydrophobic cores and hydrophilic shells) and nanoemulsions (a mixture of two immiscible liquids with surfactants) [61]. According to the size, drug carriers can be classified as implants (> 1 mm), microparticles (MPs, particles with a size ranging from 1 to 1000 µm) and nanoparticles (NPs, particles with a size less than 1000 nm) according to their size[61, 156, 157]. Currently, NPs remain the most intensively used form due to their small size, easily modified surface, ability to adsorb, attach and encapsulate various substances, and favourable biocompatibility [61, 158, 159]. According to the physical structures, drug carriers can be roughly divided into reservoir-type and matrix-type drug delivery systems [9, 156] (Fig. 4). In a reservoir-type carriers, drug agents are trapped in an inner core, surrounded by a polymer wall that controls the rate of drug release [9, 85, 105, 160]. In a matrix-type carriers, the agents are buried within and uniformly distributed throughout the polymer matrix [9, 105, 160]. Considering that glaucoma is a chronic neurodegenerative disease, an ideal anti-glaucoma DDS should be able to constantly release drugs in a zero-order fashion near the target sites [9, 105]. Researchers suggest that matrix-type systems typically release drugs at a declining rate (in a non-zero-order fashion), while reservoir-type systems release drugs at a constant rate (in a zero-order fashion) [9]. Moreover, reservoir-type delivery systems have a relatively higher loading capacity compared with matrix-type systems of the same-size [160]. In fact, patterns of drug release and total loading capacity are governed by the design and manufacture of DDS, and general trends may not be applicable to each system [9]. The release profiles can be investigated by in vitro and in vivo drug release assays. The daily release rate and cumulative release curves of drug substances under in vitro or in vivo environments can be identified by quantifying the drug substances in samples taken at different time points. The method used for quantification typically depends on the physicochemical properties of the drug cargo, such as enzyme-linked immunosorbent assay (ELISA) for protein cargo [161], and high-performance liquid chromatography (HPLC) for small molecules [127]. If drugs absorb radiation in UV light, quantification of absorption by a mass spectrometer is also a viable strategy [162].

**Fig. 5:**
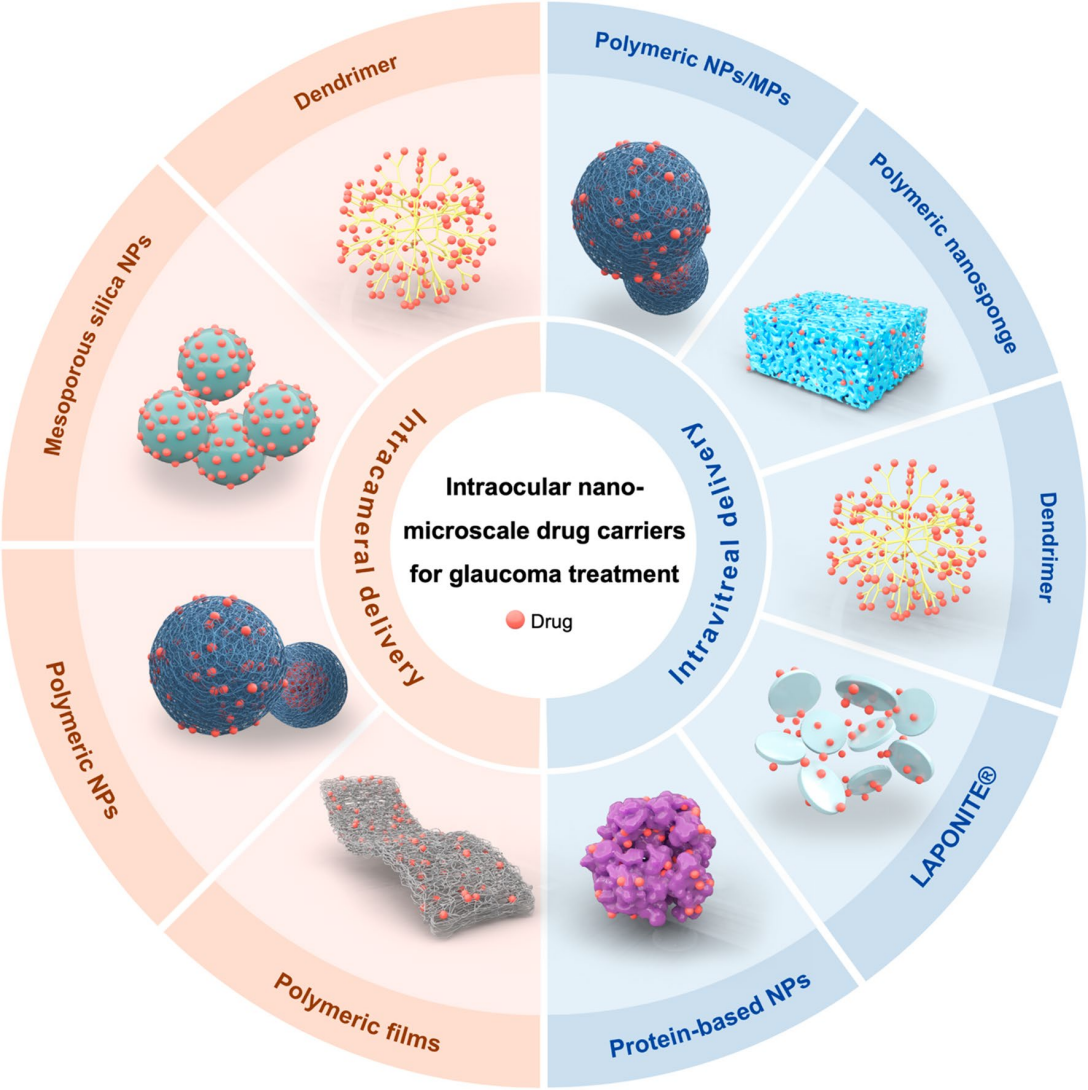
Schematic diagram of the nano-microscale carriers used to construct anti-glaucoma intraocular drug delivery systems. MPs, microparticles; NPs, nanoparticles

In conclusion, the ability to provide superior pharmacological effects, adequate biocompatibility, in vivo stability, and appropriate sterilization techniques are prerequisites for using the material as a drug carrier. The right choice of the most adequate design of DDSs depends on the target site, the drug to be delivered, and the desired drug release pattern. To improve biocompatibility or optimize the durg release patterns, it is common to incorporate different forms of materials or addictives into one hybrid system. The possibilities for the design of nanocarriers are almost infinite. To provide an overall picture, a schematic representation of the common forms and materials used to construct anti-glaucoma drug carriers in shown Fig. 5.

## Intraocular drug delivery systems under preclinical investigation

Basing on their delivery methods, intraocular drug delivery systems can be classified as intracameral and intravitreal delivery systems. They can further be classified according to the type of main materials responsible for carrying drug cargo.

### Intracameral delivery systems

#### Inorganic materials-based

**Fig. 6:**
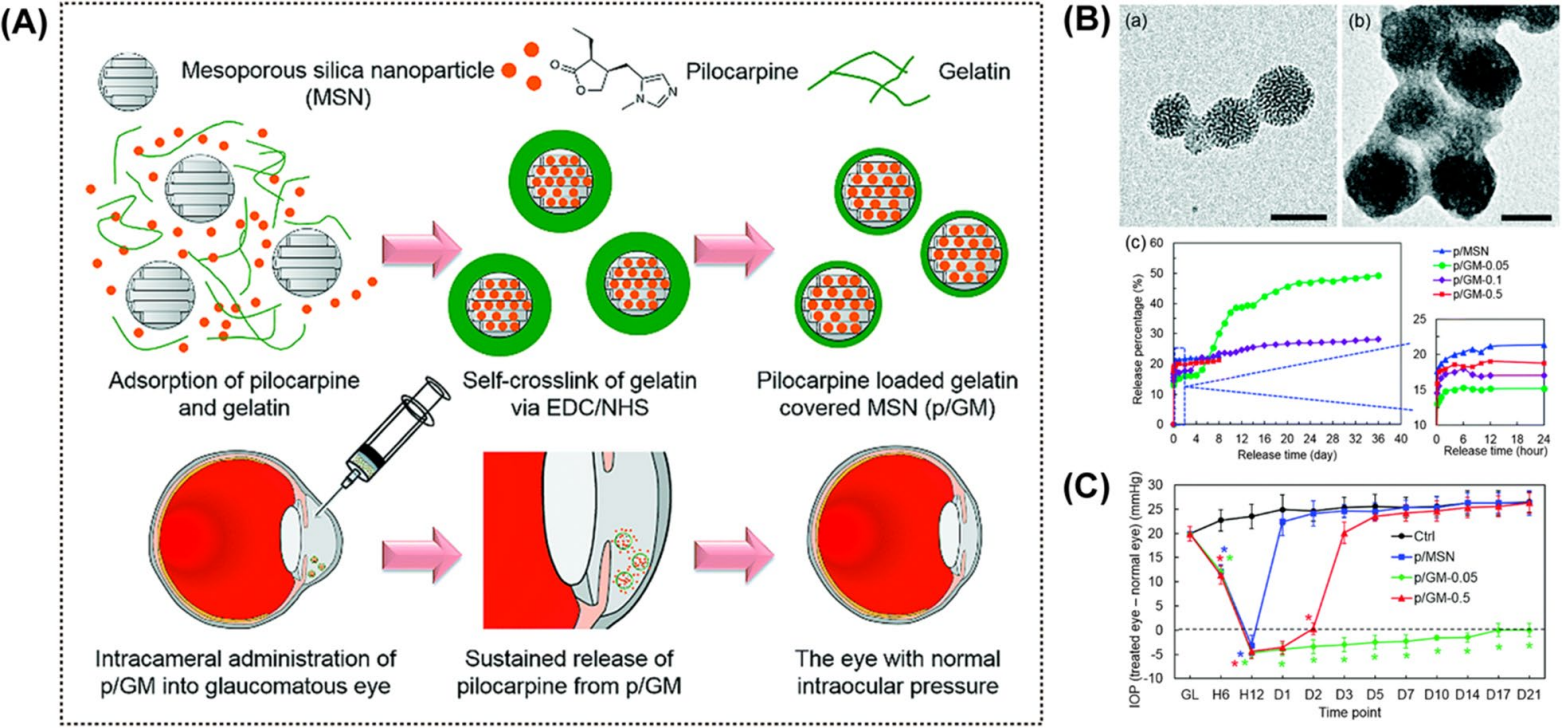
Inorganic materials-based intracameral drug delivery systems. **A** Synthesis of pilocarpine-loaded gelatin-covered MSNs (p/GNs). **B** Transmission electron microscopic images of the (a) MSNs and (b) gelatin-covered MSNs. Scale bar = 50 nm. (c) Cumulative release of pilocarpine from MSNs without gelatin coating and gelatin-covered MSNs (p/GM-x, x is denoted as mg of gelatin on per mg of MSN). **C** IOP change of the eyes treated with p/GM-x. GL: glaucomatous eyes before operation; H: hour after the administration; D: day after the administration. Reprodcued from Ref. [168] with permission from the Royal Society of Chemistry

MSNs have been proven to be excellent candidate drug carriers due to their large pore volume, high surface areas, easy surface functionalization, and low biotoxicity [163–165]. A popular drug of choice for intracameral drug delivery is pilocarpine, an agent that effectively induces ciliary muscle contraction and miosis, leading to increased outflow of aqueous humour and decreased IOP [81, 166, 167]. Liao et al. developed gelatin functionalized pilocarpine-loaded MSNs. They used gelatin as protection of drug-loaded MSNs to extend the drug release and improve ocular bioavailability, and administrated the delivery system by intracameral injection (Fig. 6A) [168]. This DDS steadily release pilocarpine after the sixth hours post-administration and released approximately 50% of the drug payload for a long time (36 days) in vitro (Fig. 6B). In the OHT rabbit eyes, this DDS reduced and maintained IOP for 21 days (Fig. 6C).

#### Organic materials-based

##### Dendrimers-based

Dendrimers are a class of polymers with well-defined radially symmetrical branched structures. They are composed of three components namely, a central core, the branches, and terminal functional groups. Dendrimers can be functionalized to cater to different requirements and this versatility makes dendrimers widely used vehicles for drug delivery [55, 169]. Among various chemistries, poly(amidoamine) (PAMAM) dendrimers are frequently used for ocular applications.

**Fig. 7:**
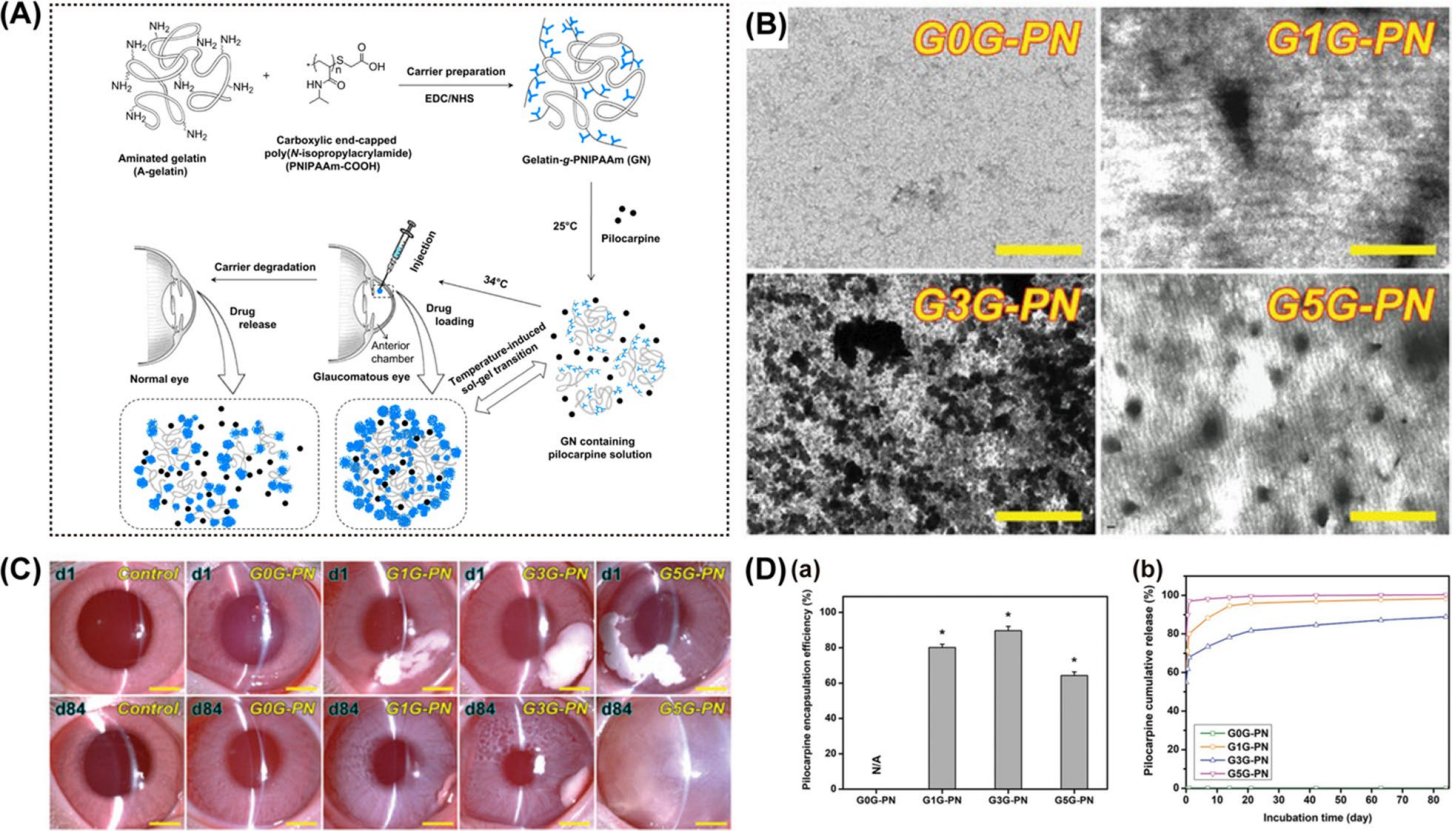
Dendrimer-based intracameral drug delivery systems. **A** Schematic representation of the manufacture of gelatin grafted PNIPAAm (gelatin-g-PNIPAAm) and intracameral administration of pilocarpine-loaded gelatin-g-PNIPAAm. Reprinted from Ref. [166] with permission from Elsevier. **B** Transmission electron microscopic images of PAMAM tethered gelatin-g-PNIPAAm (GxG-PN) (x is denoted as the percentages of amino groups in gelatin samples). **C** Representative slit-lamp biomicroscopic images of rabbit eyes at day 1 and 84 after intracameral injection of GxG-PN thermogels. Scale bars = 5 mm. **D** The encapsulation efficiency of GxG-PN (a) and cumulative release percentage of pilocarpine from GxG-PN (b). (B)—(D): Reprodcued from Ref. [167] with permission from ﻿WILEY–VCH Verlag GmbH and Co. KGaA, Weinheim

Lai et al. formulated pilocarpine-loaded gelatin grafted poly(N-isopropyl acrylamide) (PNIPAA) thermogel with a high encapsulation efficiency (around 62%) (Fig. 7A). Researchers administrated this nanosystem into the anterior chamber of rabbits with ocular hypertension (OHT) with a 30-gauge needle [166]. Increased concentration of the drug in the anterior chamber and reduction of IOP continued for 2 weeks. In in vitro drug release study, ﻿the cumulative release of pilocarpine approached 95% of the payload after 14 days. Later, they used PAMAM dendrimers as a tether to bond gelatin and PNIPAA, intending to improve biodegradation resistance, drug encapsulation efficiency, and release performance [167] (Fig. 7B and C). In this study, pilocarpine and ascorbic acid were encapsulated in the carrier at the same time. After the intracameral injection of a single dose of the DDS (10% w/v), reduced IOP lasted over 80 days (Fig. 7D). This DDS also showed multifunctional abilities, such as attenuation of inflammatory mediators and stimulation of stromal collagen regeneration.

##### Poly (ε-caprolactone)-based

**Fig. 8:**
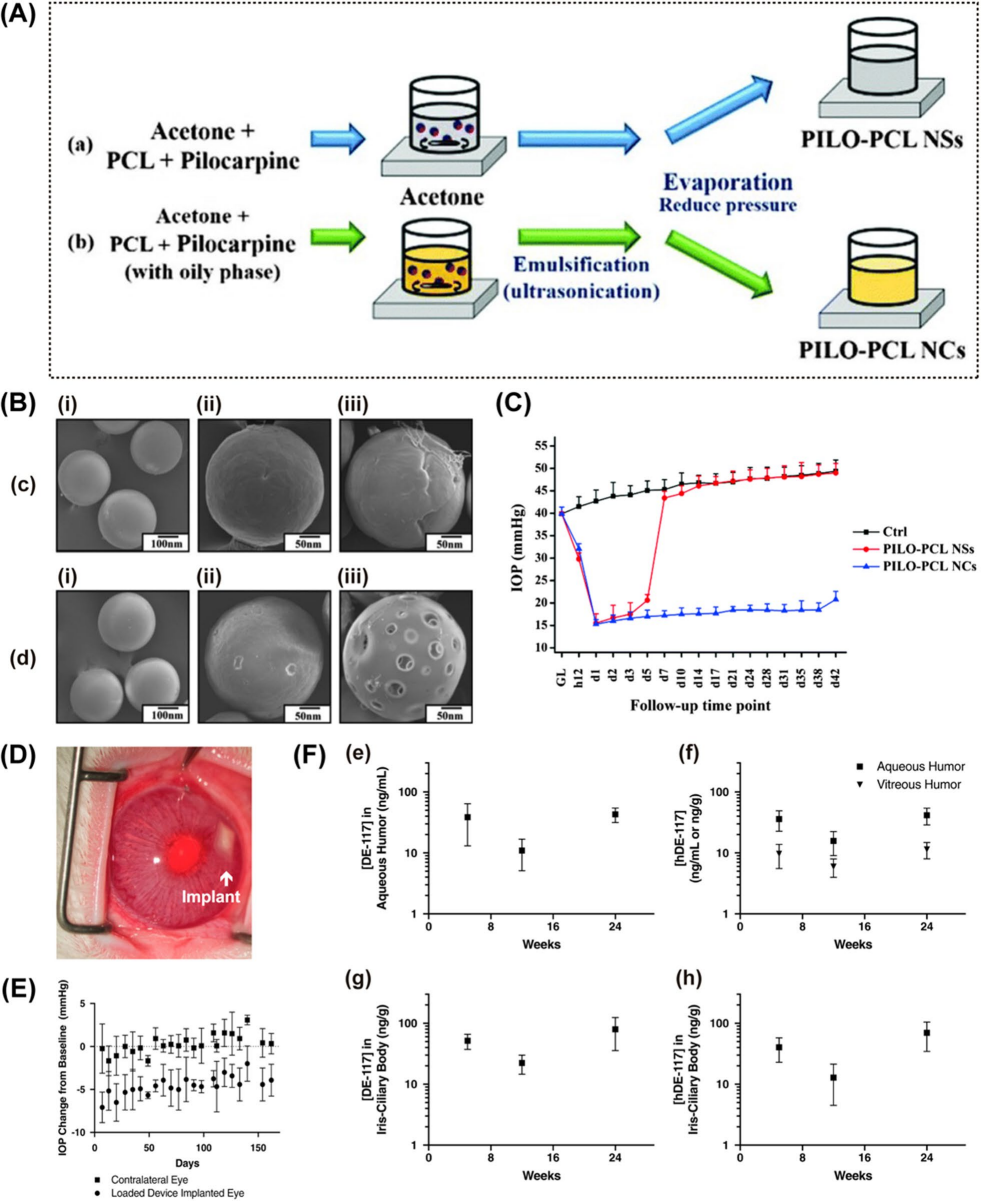
Poly (ε-caprolactone)-based intracameral drug delivery systems. **A** Schematic representation of the synthesis of pilocarpine-loaded PCL nanospheres (NSs) (a) and PCL nanocapsules (NCs) (b) via the emulsion-solvent evaporation method. **B** Scanning electron microscopic images of (c) pilocarpine-loaded NSs (PILO-PCL NSs) and (d) pilocarpine-loaded NCs (PILO-PCL NCs) dispersed in BSS buffer (i) and at day 42 (ii) and day 70 (iii). **C** IOP change of glaucomatous rabbit eyes treated with 20 μL of PILO-PCL NSs or PILO-PCL NCs. Follow-up time point: day (d). **D** Representative photograph after device implantation. **E** IOP changes of the eyes after DE-117-loaded device implantation. **E** Concentration of DE-117 and hDE-117 (active form of DE-117) in the aqueous humor and vitreous (e, f) and iris-ciliary body (g, h) at different time points after the administration of DE-117-loaded implant. **A**—**C** Reproduced from Ref. [160] with permission from the Royal Society of Chemistry. (D)—(F): Reprinted from Ref. [123] with permission from Elsevier

Poly (ε-caprolactone) (PCL) exhibits satisfactory bioresorbable and biodegradable profiles in living tissues, and it can be easily manufactured into various shapes such as thin films or capsules [55]. Lee et al. synthesized two types of pilocarpine-loaded PCL NPs via the emulsion-solvent evaporation method (nanospheres and nanocapsules) and compared their drug release patterns as well as their in vivo treatment effects [160] (Fig. 8A and B). In this study, PCL nanocapsules show better (approximately 3 times higher) loading capacity than the nanospheres. The release of pilocarpine from the nanocapsules lasted up to 40 days, while about 85% of pilocarpine has been released from the nanospheres on day 6. In OHT rabbit eyes, ﻿intracamerally injected pilocarpine-loaded PCL nanocapsules succussed to reduce and maintain the ﻿IOP to ﻿below 20 mmHg for 42 days, while pilocarpine-loaded PCL nanospheres failed to maintain IOP reduction effect after day 7 post-administration (Fig. 8C). In this work, it seems the drug-carrying PCL nanocapsules own greater therapeutic potential compared with drug-carrying PCL nanospheres.

There are relatively few studies of intracameral implants. Kim et al. developed DE-117 loading intracameral implants based on PCL films [123, 170]. DE-117 is a novel hypotensive agent that has been proven to activate the EP2 receptor and increase the trabecular outflow, which may be an alternative for patients who are unresponsive to conventional PGA eye drops [13, 171, 172]. In 2016, this team generated biodegradable DE-117 contained PCL films using spin-casting techniques [170]. The final drug-loaded PCL implant of approximately 3 × 3 mm in size was implanted into the anterior chamber of normotensive rabbit eyes through a 4-mm corneal incision. Good tolerance of the implant was observed during the follow-up period. In vitro drug release profiles demonstrated a release rate of 0.5 µg/day for more than 6 months. Based on these findings, the researchers later optimized the device. The newly developed DE-117 loaded PCL implant had a smaller size (2.5–3 mm in width and length; 180 μm in thickness) (Fig. 8D). It is estimated that each device contains 146 ± 79 µg DE-117 and releases DE-117 at a rate of 0.49 ± 0.11 µg/day. A 3-mm corneal incision made by a slit knife was used to insert the implant into the anterior chamber of the rabbit eye. After ﻿device implantation, reduced IOP was observed for 23 weeks (Fig. 8E). Additionally, in vivo drug distribution analysis showed that the concentration of DE-117 (and its hydrolyzed form) was maintained for up to 24 weeks in target tissues (both the aqueous humour and the iris-ciliary body) [123] (Fig. 8F).

##### Poly (lactic acid)-based

**Fig. 9:**
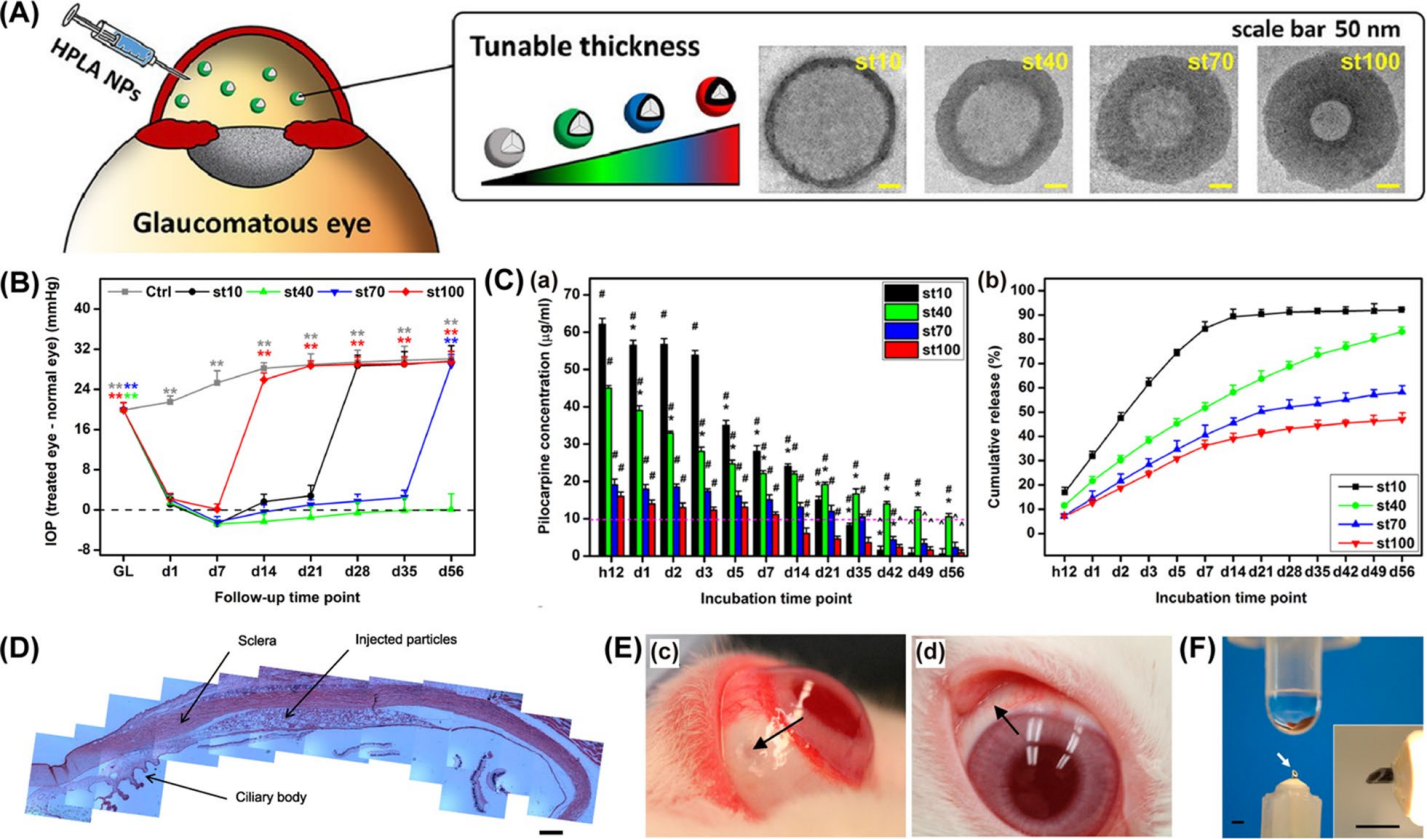
Poly (lactic acid)-based intraocular drug delivery systems. **A** Transmission electron microscopic images of pilocarpine-loaded hollow PLA (HPLA) NPs with tunable shell thickness and intracameral injection of the NPs. **B** IOP change of the eyes treated with different pilocarpine-loaded HPLA NPs. Follow-up time point: day (d). **C** The concentration of released pilocarpine (a) and cumulative release percentage (b) of pilocarpine from different types of pilocarpine-loaded HPLA NPs in in vitro drug release assays. Incubation time point: hour (h); day (d). **D** Histological images of the rabbit eye at day 46 after supraciliary injection of brimonidine-loaded microspheres. Scale bar = 500 μm. **E** Representative photographs of the rabbit eye 5 min (c) and 1 day (d) after supraciliary injection. Arrows: the injection sites. **F** The photographs of a microneedle fabricated in this study (compared with a 50 μL drop from a conventional eye dropper). Scale bars = 1 mm. **A**—**C** Reprinted from Ref. [81] with permission from Elsevier. **D**—**F** Reprinted from Ref. [84] with permission from Elsevier

Poly (lactic acid) (PLA) is an eco-friendly, hydrophobic, thermoplastic material [55]. ﻿Nguyen et al. [81] compared the attributes of pilocarpine-loaded PLA nanocapsules with different shell thickness ranging from 10 to 100 nm (Fig. 9A). The study showed that a single intracameral injection of 20-μL median thick shells (approximately 40 nm) NPs allowed a therapeutic drug release in OHT rabbit eyes for 56 days and provided the protections of retinal structures and visual functions (Fig. 9B). Furthermore, NPs with 40-nm shells demonstrated a steady drug release profile in drug release analysis. No burst release was observed during the analysis. At the endpoint of the analysis (day 56), about 80% of the drug payload can be released from the NPs. NPs with thicker shells (approximately 70–100 nm) show relatively low loading capacity and NPs with thin shells (approximately 10 nm) provide poor sustained release performance (Fig. 9C).

### Intravitreal delivery systems

#### Organic materials-based

##### Poly (lactic acid)-based

The supraciliary route delivery means that the needle enters close to the ciliary body and the final drug deposition is mainly above the ciliary body (for the review on the supraciliary spaces for drug delivery, refer to [173]). Supraciliary brimonidine with a lower dose (0.75 µg) was found to provide an equal magnitude and duration of IOP reduction compared with brimonidine eye drops (75 µg), indicating the superior bioavailability of brimonidine via supraciliary administration [174]. Subsequently, Chiang et al. administrated brimonidine-loaded PLA microspheres into multiple locations of ﻿the supraciliary space of rabbit eyes with a designed 27-gauge hypodermic needle [84] (Fig. 9D and E). This microneedle was shortened to match the thickness of the sclera and conjunctiva (with a total length of 750 ± 50 μm) (Fig. 9F). After a single dose of drug-loaded microspheres (30 mg, containing 0.9 mg brimonidine), IOP was reduced (by progressively smaller amounts) for 33 days. In in vitro drug release analysis, incomplete drug release (less than 80% of the payload) was observed till the end of the study (five weeks). This study demonstrated the therapeutic potential of a highly-targeted delivery method. However, multiple injections were required to complete a single dose of the DDS, and the drug release pattern of the DDS may need to be optimized.

##### Poly (lactic-co-glycolic acid)-based

**Fig. 10:**
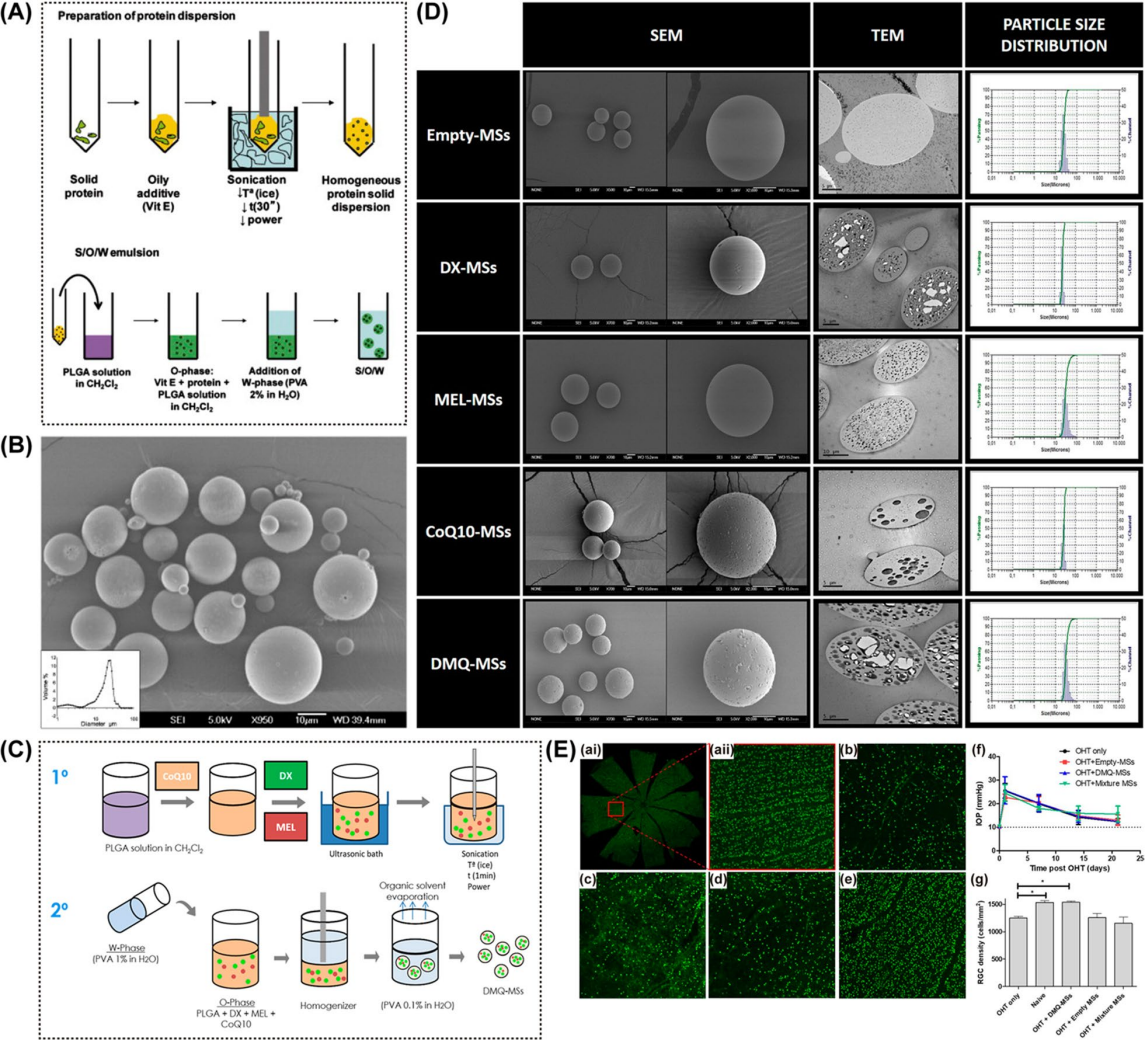
Poly (lactic-co-glycolic acid)-based intravitreal drug delivery systems. **A** The scheme of microspheres elaboration via the novel solid-in-oil-in-water (S/O/W) method. **B** The scanning electron microscopic image of GDNF-loaded microspheres. **C** Elaboration process of the examethasone/melatonin/coenzyme Q10-loaded PLGA microspheres (DMQ-MSs). **D** Scanning and transmission electron microscopic images of empty MSs and drug-loaded MSs. Particle size distributions of different MSs are also presented. (E) The density of RGCs in naïve retinas (ai and aii), untreated retinas (b), empty MSs-treated retinas (c), mixture MSs-treated retinas (d), and DMQ-MSs-treated retinas. Each red box = 1 mm^2^. (f) and (g) present statistical analyses of IOP change and RGCs density respectively after different treatments on ocular hypertension models. **A**, **B** Reprinted from Ref. [102] with permission from Elsevier. **C**—**E** Reprinted from Ref. [179] with permission from Elsevier

PLGA is a synthetic biodegradable polyester approved by the Food and Drug Administration (FDA) of the United States for human applications. It is synthesized through random ring-opening copolymerization of the cyclic dimers of glycolic acid and lactic acid [55]. The degradation duration of PLGA can range from weeks to years and is largely dictated by the ratio of glycolic acid and lactic acid [55]. Several research teams have evaluated the glial cell line-derived neurotrophic factor (GDNF)—loaded PLGA NPs for the neuroprotection of glaucoma. GDNF is widely expressed in the central nervous system and has been proven to be beneficial for RGC survival [18, 175, 176]. Using DBA/2J mice, Ward et al. demonstrated that the intravitreal injections of GDNF-loaded PLGA microspheres resulted in the cumulative release of GDNF for more than 71 days, with 3.5 times greater RGC density than the untreated group (at 15 months) [177]. Similar neuroprotective effects of the GDNF-loaded DDS have also been demonstrated successfully in Morrison’s OHT rat models [178]. Later, Checa-Casalengua et al. utilized PLGA microspheres as carriers for both GDNF and vitamin E (25.4 ng GDNF/mg particles) [102] (Fig. 10A and B). In OHT rat models, a single dose of GDNF + vitamin E-loaded PLGA NPs (﻿﻿0.64 ng GDNF/eye) delivery by an intravitral injection demonstrated approximately two times greater protection of RGC and its axon compared with GDNF, vitamin E or blank microspheres alone. In vitro drug release study proved that GDNF was released from the microspheres in a sustained fashion until the end of the assay (day 133). The possibility of jointly releasing several different substances with a single drug carrier is explored by Arranz-Romera et al. [179]. They developed PLGA microspheres (loading effiency 72.99 ± 0.60%) loaded with dexamethasone, melatonin, and coenzyme Q10 to achieve simultaneous co-delivery (Fig. 10C). The microspheres have a homogeneous particle size of 29.04 ± 1.89 μm, as a property that renders them injectable with conventional 25 – 32 gauge needles (Fig. 10D). Drug release studies confirmed that the three drug agents were continuously released from the loaded microspheres for up to 30 days, but with different release patterns. After the initial burst release effect, dexamethasone was released at a rate of 0.60 ± 0.04 μg/mg microspheres/day until day 24 and ﻿1.20 ± 0.15 μg/ mg microspheres/day untill day 30. Melatonin was released at a rate of 1.66 ± 0.31 μg/mg microspheres/ day until day 14 and 0.69 ± 0.18 μg/mg microspheres/day the endpoint of the study. The release rate of coenzyme Q10 was steady through the study period,which was 0.63 μg/mg microspheres/day, In vivo efficacy studies using Morrison’s OHT rat models showed that an intravitreal injection (2.5% w/v, via a 30-gauge hypodermic needle) of multi-loaded microspheres (containing 11.5 μg dexamethasone + 4.6 μg melatonin + 3.6 μg coenzyme Q10) preserved retinal structures and functions 21 days after administration; in contrast, ﻿the equivalent individual single-drug microspheres and empty microspheres showed no such protective effect (Fig. 10E).

##### Dendrimers-based

**Fig. 11:**
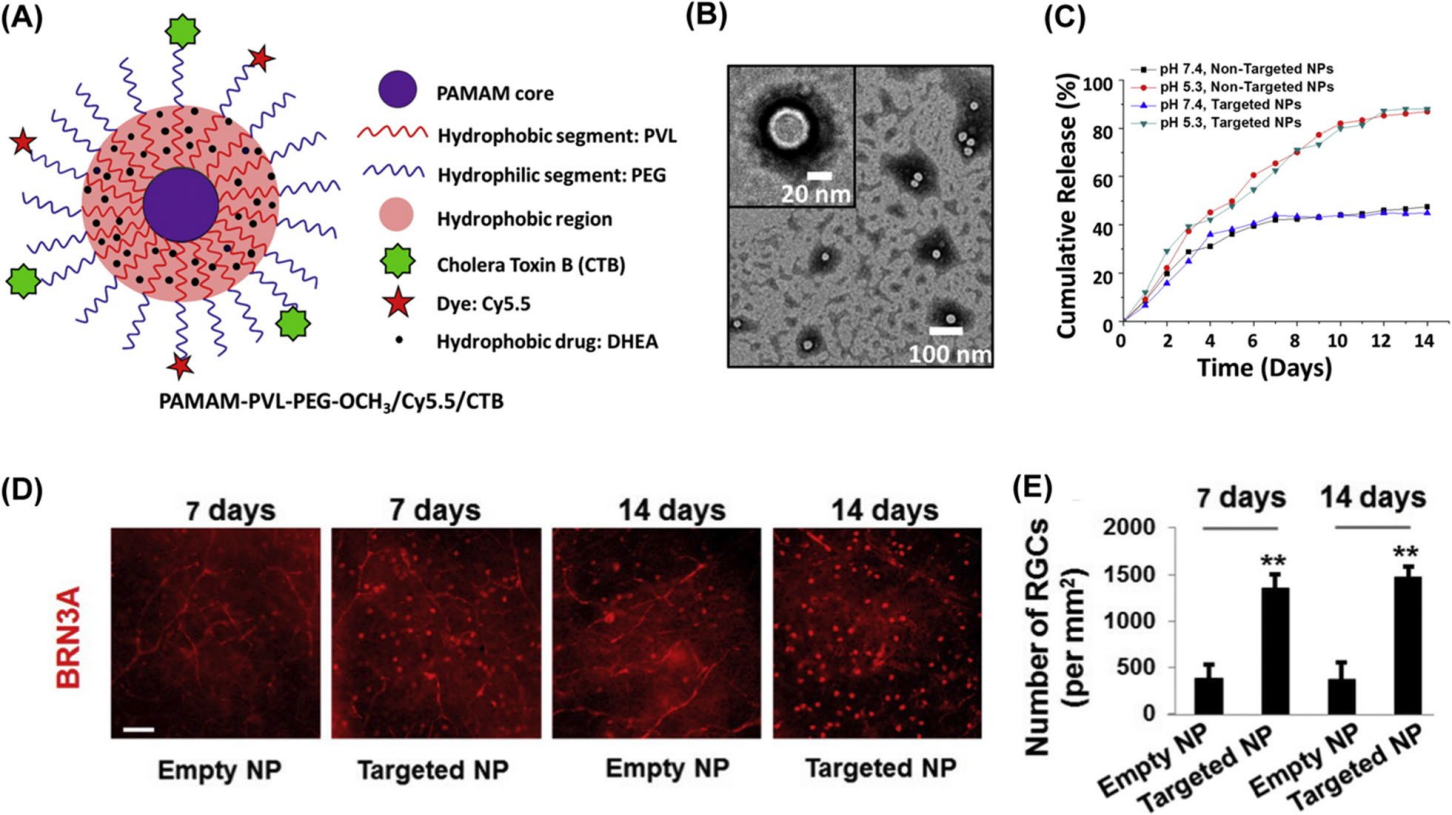
Dendrimers-based intravitral delivery system. **A** A schematic illustration of the manufacture of copolymer nanoparticles (unimNPs) conjugated with the RGC-targeting CTB (CTB-unimNPs) for target delivery of DHEA to the RGCs. **B** Transmission electron microscopic images of the unimNPs. **C** In vitro DHEA release profiles from DHEA-loaded non-targeted (unimNPs) and targeted NPs (CTB-unimNPs). **D** Representative images showing RGCs density (BRN3A-positive) in the retinas collected at day 7 and day 14 after intravitreal injection of CTB-unimNPs or DHEA-loaded CTB-unimNPs. Scale bar = 100 μm. **E** Statistical analyses of RGCs amount in the retinas treated by CTB-unimNPs and DHEA-loaded CTB-unimNPs. Reprinted from Ref. [180] with permission from Elsevier

RGC-targeted drug delivery was observed with NPs formed by multi-arm star amphiphilic block copolymer (poly (amidoamine)-polyvalerolactone-poly (-ethylene glycol), PAMAM-PVL-PEG) [180]. The researchers conjugated NPs with the cholera toxin B domain (CTB) for RGC targeting and dehydroepiandrosterone (DHEA) (a sigma-1 receptor agonist) for therapeutic effects (Fig. 11A and B). Over 2 months release of DHEA was observed during in vitro drug release analysis. At day 14 of the analysis, a total of less than 50% of DHEA payload was released from the targeted NPs (Fig. 11C). In vivo neuroprotective treatment outcome was evaluated using RGC degeneration mice models. After a single intravitreal injection of the CTB-conjugated NPs (2 μL, containing 0.5 μg DHEA and 2 μg NPs), the NPs accumulated in the RGC layer and provided RGCs preservation for up to 2 weeks. On the contrast, NPs without CTB showed barely accumulation or protection of RGCs in vivo (Fig. 11D and E).

##### Protein-based

Other than its IOP reduction effect, brimonidine can also provide protections to the RGCs. The neuroprotective effects of brimonidine-loaded DDSs were investigated on normotensive models with RGCs degeneration. Kim et al. fabricated human serum albumin NPs (HSA-NPs) containing 0.18% brimonidine (HSA-Br-NP). They used rat eyes with optic nerve crush (ONC) as animal models. At day 14 after the intravitreal injection of the HSA-Br-NP, treated group showed significantly higher RGC density than the sham group. In addition, they found that retinas treated with HSA-NPs (containing no brimonidine) also exhibited increased RGCs survival and reduced amyloid-β deposition in the RGC layer compared with untreated eyes, which indicated the additional therapeutic effect of the carrier materials themselves [181].

#### Inorganic materials-based

LAPONITE^®^ are biodegradable synthetic clays composed of two-dimensional disk-shaped crystals that attract surrounding molecules. Brimonidine-loaded LAPONITE^®^ formulation was created by Rodrigo et al.[82] Brimonidine-LAPONITE^®^ lowered IOP for up to 56 days and released brimonidine for up to 6 months. Treated OHT eyes also exhibited better retinal structural integrity than untreated eyes in optical coherence tomography (OCT) exams and immunohistochemistry assays.

**Fig. 12:**
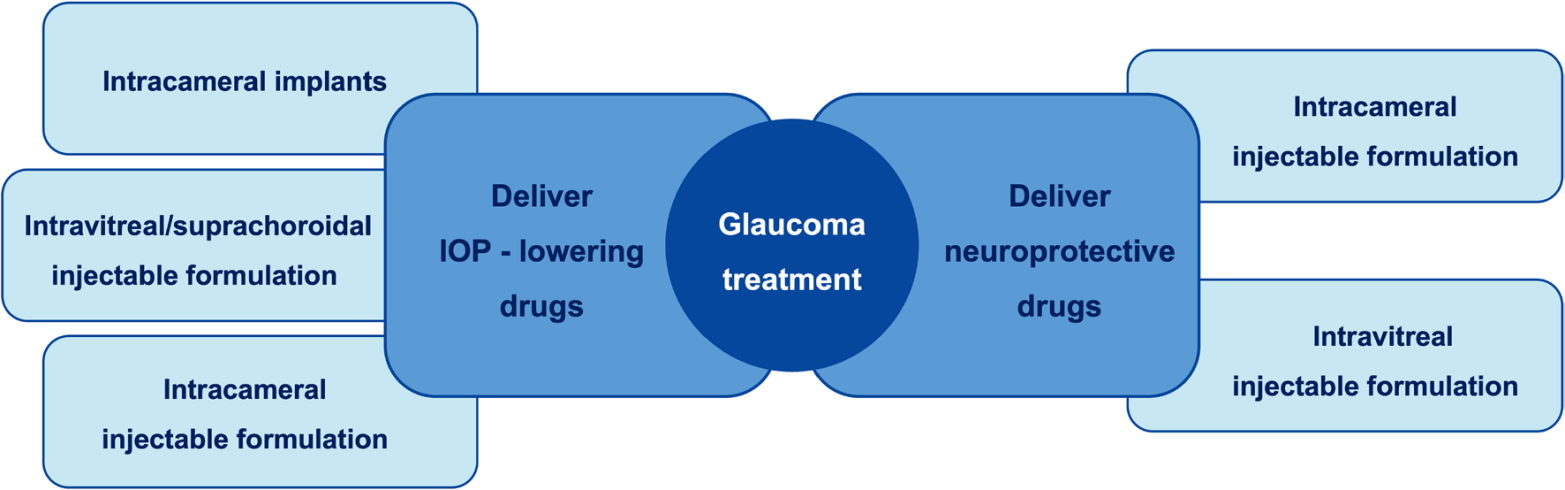
Schematic diagram of intraocular drug delivery systems used to deliver anti-glaucoma drugs. IOP, intraocular pressure

Section 3 reviews various studies of intraocular nanoscale drug carries-based therapies using glaucomatous animal models. A summary of studies reported over the past two decades is presented in Fig. 12 and Table 2.

**Table 2:** In vivo studies on intraocular drug delivery systems targeting glaucomatous symptoms (data accessed in February of 2023)

Year	Drug carriers	Drug delivered	Route	Animal model	Main in vivo effect	References
2006	PLGA nanospheres	PEDF peptide	IVJ	Retinal I/R injury mice	Sustained release	[182]
2007	PLGA microspheres	GDNF	IVJ	DBA/2 J mice	Sustained release	[177]
2007	PLGA microspheres	GDNF	IVJ	OHT rat	Sustained release	[178]
2011	PLGA microspheres	GDNF + vitamin E	IVJ	OHT rat	Sustained release	[102]
2011	Gelatin-grafted-PNIPAAm	Pilocarpine	Intracameral injection	OHT rabbit	Thermo- responsive; sustained release	[166]
2011	﻿PLGA microspheres & nanospheres	AG1478	IVJ	ONC rat	Sustained release	[146]
2011	Poly (γ-glutamic acid) NPs	Dexamethasone	IVJ	NMDA induced retinal injury rat	Sustained release	[183]
2015	HSA NPs	Brimonidine	IVJ	ONC rat	Sustained release	[181]
2015	PLGA NPs and MPs	Connexin43 peptide	IVJ	Retinal I/R injury rat	Sustained release	[184]
2015	Nanosponge	Brimonidine; Travoprost; Bimatoprost	IVJ	OHT mice	Sustained release	[185]
2016	PCL	DE-117	Intracameral delivery	Normal rabbit	Sustained release	[170]
2016	PLA microspheres	Brimonidine	Supraciliary delivery	Normal rabbit	Sustained release	[84]
2017	PAMAM–PVL–PEG	Dehydroepiandrosterone	IVJ	NMDA induced retinal injury mice	Target delivery; sustained release	[180]
2017	Gelatin-coated MSNs	Pilocarpine	Intracameral injection	OHT rabbit	Sustained release	[168]
2017	GA-grafted-gelatin-PNIPAAm	Pilocarpine	Intracameral injection	OHT rabbit	Thermo- responsive; sustained release	[186]
2017	PCL NPs	Pilocarpine	Intracameral injection	OHT rabbit	Sustained release	[160]
2018	PCL	DE-117	Intracameral delivery	Normal rabbit	Sustained release	[123, 170]
2018	PEG-PSA MPs	Dorzolamide	IVJ	OHT rat	Sustained release	[187]
2018	HA-coated HSA NPs	Connexin43 peptide	IVJ	Retinal I/R injury rat	Sustained release	[188]
2018	﻿Sul − PSHU − PNIPAAm	CNTF	IVJ	ONC rat	Thermo- responsive; sustained release	[189]
2019	PLGA microspheres	Dexamethasone + melatonin + coenzyme Q10	IVJ	OHT rat	Sustained release	[179]
2019	Chitosan-grafted- PNIPAAm	Pilocarpine + RGFP966	Intracameral injection	OHT rabbit	Thermo- responsive; sustained release	[190]
2019	PAMAM-gelatin-grafted -PNIPAAm	Pilocarpine + ascorbic acid	Intracameral injection	OHT rabbit	Thermo- responsive; sustained release	[167]
2020	LAPONITE®	Brimonidine	IVJ	OHT rat	Sustained release	[82]
2020	PAMAM	Superoxide dismutase	IVJ	Retinal I/R injury rat	﻿Intracellular delivery	[191]
2020	Hollow PLA NPs	Pilocarpine	Intracameral injection	OHT rabbit	Sustained release	[81]
2020	PEG400	Tafluprost	IVJ	ONT rat	Sustained release	[192]
2020	PEG-PSA NPs	Brinzolamide + miRNA-124	IVJ	ONC mice	Sustained release	[193]
2021	﻿Polydopamine NPs	﻿Brimonidine	IVJ	ONC mice	Sustained release	[194]

PLGA, poly (lactic-co-glycolic acid); PEDF, pigment epithelium-derived factor; IVJ, intravitreal injection; I/R, ischemia–reperfusion; GDNF, glial cell line-derived neurotrophic factor; OHT, ocular hypertension; PNIPAAm, poly (N-isopropylacrylamide); AG1478, ﻿4-(3-chloroanilino)-6,7-dimethoxyquinazoline (a tyrosine kinase inhibitor of the e﻿pidermal growth factor receptor); ONC, optic nerve crush; NPs, nanoparticles; NMDA, N-methyl-D-aspartate; ﻿﻿HSA, human serum albumin; MPs, microparticles; PCL, poly (ε-caprolactone); PLA, poly (lactic acid); PAMAM, poly (amidoamine); PVL, polyvalerolactone; PEG, polyethylene glycol; HA, hyaluronic acid; Sul − PSHU − PNIPAAm, sulfonate functionalized poly (serinol hexamethylene urea) (PSHU) conjugated PNIPAAm; CNTF, ﻿cilliary neurotrophic factor; MSNs, mesoporous silica nanoparticles; GA, gallic acid; PEG-PSA, Poly (ethylene glycol)-co-poly (sebacic acid); ONT, optic nerve transection

## Intraocular drug delivery systems under clinical investigation

To date, the Durysta™ bimatoprost sustained-release (SR) intracameral implant by Allergan is the only government regulatory agency-approved sustained-release therapy for glaucoma [195]. Other intraocular DDSs therapies that have been clinically investigated among human patients include iDose^®^ TR ﻿(Glaukos Corporation, San Clemente, CA, USA), OTX-TIC (Ocular Therapeutix, Bedford, MA, USA), PA5108 latanoprost free acid sustained-release implant (PolyActiva Pty Ltd, Parkville, Australia), ENV515 Travoprost Extended Release (XR) (Envisia Therapeutics, Durham, NC, USA) and NT-501 implant (Neurotech Pharmaceuticals, Inc., Cumberland, RI, USA). Well-established and complete clinical trials involving human patients are of considerable importance before laboratory-to-bedside translation, along with the occurrence of commercialization.

### Durysta

Durysta™ (Bimatoprost SR) is a biodegradable intracameral implant based on the PLGA matrix Novadur^®^ platform. Durysta™ contains 10 μg of bimatoprost for IOP reduction in patients with open-angle glaucoma (OAG) or OHT [141]. The implant is designed to be injected into the anterior chamber via a single-use prefilled applicator containing a 28-gauge needle, intending to release the bimatoprost for 4–6 months in a non-pulsatile, zero-order kinetic fashion [195–200].

During the preclinical investigation period, Bimatoprost SR showed an equal or superior IOP reduction effect for up to 66 days compared with the once-daily dose of 0.03% bimatoprost eye drops on beagle dogs [197, 198]. Additionally, off-target phenomena were rarely observed in extraocular tissues, suggesting that the implant reduced the risk of common side effects with conventional topical PGA eye drops [199].

In the Bimatoprost SR phase I/II clinical trial (24-month, dose-ranging, paired-eye controlled), over 70 OAG patients were administered intracamerally with Bimatoprost SR (a single dose, 6 μg, 10 μg, 15 μg, or 20 μg) in one eye, while the fellow eye was administrated 0.03% topical bimatoprost once daily. Bimatoprost SR demonstrated good tolerance throughout the trial [196, 200]. Favourable IOP control was observed in most patients for up to 6 months, and up to 40% and 28% of patients without any additional interventionmaintained IOP for up to 1 and 2 years, respectively [196, 200]. The most commonly reported immediate post-administration (within 2 days after the administration) ocular AE is conjunctival hyperemia, which may be associated with the application of ophthalmic povidone-iodine. Beyond the immediate post-administration period, the overall incidence of AEs was equivalent between implant-administrated eyes and eye drops-applied eyes. AEs typically with topical PGA, such as orbital fat atrophy and eyelash growth, were lower in the eyes treated with the implant than those treated with topical bimatoprost [196]. In 2020, Allergan received approval of using the Durysta™ Bimatoprost SR (10 μg) implant for intracameral administration to treat OAG or OHT [195, 201, 202]. Durysta™ is limited to a single implant per eye with no further treatment, and caution must be paid in patients with limited corneal endothelial cell reserve or narrow iridocorneal angles (Shaffer grade < 3) [141]. Currently, Allergan continues Phase III studies with Durysta™ to power further label enhancement and approvals of other countries [142].

### iDose

iDose^®^ TR ﻿(Glaukos Corporation, San Clemente, CA, USA) is a non-biodegradable titanium intracameral implant with a built-in membrane containing travoprost [11, 13]. This device is designed to be placed ﻿in the nasal trabecular meshwork with its scleral anchor [11, 13]. iDose® TR provides ﻿therapeutic levels of travoprost for at least one year and once all cargo runs out, the implant will be removed and replaced with a new one [203]. In its ﻿multi-centre, double-blind Phase II clinical trial, this device was found to provide 7.4–7.9 mmHg IOP reduction at 24 months and ﻿sustain IOP control for more than 36 months [203, 204]. Currently, Phase III studies randomized a total of 1,150 subjects covering over 80 sites in the United States are ongoing and are expected to support ﻿FDA approval for iDose^®^ TR in 2023 [203, 205].

### OTX-TIC

OTX-TIC (Ocular Therapeutix, Bedford, MA, USA) is a biodegradable intracameral implant containing a travoprost-loaded microparticle-embedded hydrogel matrix [206–208]. This device is designed to release travoprost in the anterior chamber for 4–6 months [206]. Recent reports have shown that OTX-TIC provides equivalent treatment outcomes in IOP reduction compared with topical travoprost, which has persisted for up to 6 months in some patients [207, 208]. Therefore, a Phase II prospective, multi-centre, randomized, parallel-group, controlled study involving approximately 105 subjects and up to 20 sites in the United States is performed to further determine the efficacy and safety of OTX-TIC (NCT05335122). In this study, subjects are expected to be randomized into one of three treatment groups: (1) low dose of OTX-TIC; (2) high dose of OTX-TIC; or (3) a single administration of 10 μg Durysta™.

### PA5108

PA5108 is a biodegradable intracameral implant developed by PolyActiva Pty Ltd (Parkville, Australia). This implant contains latanoprost free acid-loaded polytriazole hydrogel [209], which is designed to provide a daily therapeutic dose of the drug for at least 6 months [210]. The initial safety and tolerability study of the implant has been completed (NCT03604328). Currently, a multi-centre, open-label, interventional, comparative study plays an effective role in identifying a safe and efficacious dose of latanoprost free acid in PA5108 (14.7–35.5 μg) for POAG adults is active (NCT04060758). Recently, PolyActiva announced their findings in the Phase IIa study [211]: the implant was generally well tolerated and no severe AE related to the product was observed. At least 20% of IOP reduction was observed in the low-dose cohort of PA5108 implant treatment. Additionally, complete implant biodegradation was completed at week 40.

### ENV515

ENV515 Travoprost XR is another biodegradable intracameral polymer implant developed by Envisia Therapeutics (﻿Durham, NC, USA) with PRINT^®^ technology. Its IOP-lowering effect is comparable to that of ﻿pre-study topical PGA (latanoprost, Xalatan^®^) and bimatoprost (Lumigan^®^), as well as 0.5% timolol maleate eye drops once daily. The IOP reduction from baseline was averaged to 6.7 ± 3.7 mmHg over 11 months after a single administration of the implant. No severe AE was observed in the study and the most common AE reported was early-onset hyperemia or eye redness, but these events appeared to be all transient and associated with the administration procedure [212, 213]. Unfortunately, this project seems discontinued and no public update is available in recent years [209].

### NT-501

NT-501 (or Renexus^®^ device, Neurotech Pharmaceuticals, Inc.) is an intravitreal polymeric implant containing genetically modified mammalian cells that constantly secrete the ciliary neurotrophic factor (CNTF) [214, 215]. The device consists of a polymer membrane, a sealant and a titanium anchor [8, 214, 215], which is designed to deliver therapeutic proteins for up to 24 months in a near zero-order fashion [216]. NT-501 has been intensively investigated for the treatment of dry age-related macular degeneration, retinitis pigmentosa and type 2 macular telangiectasia [9, 214, 215, 217]. Since CNTF is proven to be neuroprotective in glaucomatous eyes [218], trials involving glaucoma patients have been conducted in recent years. The initial Phase I safety study of NT-501 has been completed for glaucoma patients (NCT01408472). Two Phase II studies are ongoing: (1) NCT02862938: a randomized, sham-controlled, masked study involving 54 glaucoma patients to determine the efficacy of NT-501 implant (mainly with visual field parameters); (2) NCT04577300: a randomized, sham-controlled, masked study involving up to 30 glaucomatous eyes. The study eyes will receive 1 or 2 implants or sham surgery, and the results are pending.

Herein, the products experiencing clinical investigation and their corresponding trials are summarized and shown in Table 3.

**Table 3:** Intraocular drug delivery systems targeting glaucomatous symptoms under clinical investigations (data accessed in February of 2023)

Drug delivered	Manufacturer	Product name	Main drug carrier	Route and designed duration	Investigated condition(s)	Phase	References and ClinicalTrials.gov Identifier
Bimatoprost	Allergan plc (Dublin, Ireland)	﻿Durysta	PLGA (Novadur^®^)	Intracameral delivery; 4–6 months	OAG; OHT	Phase III (FDA approved)	[142, 195–200, 202] NCT04647214 NCT05338606 NCT03891446
Travoprost	Glaukos Corporation (San Clemente, CA, USA)	iDose	﻿Titanium and a sustained release membrane	Intracameral delivery; Over 12 months	OAG; OHT	Phase III	﻿[203–205] NCT02754596 ﻿NCT03868124
Travoprost	Ocular Therapeutix Inc (Bedford, MA, USA)	OTX-TIC	Microparticles embedded hydrogel	Intracameral delivery; 4–6 months	OAG; OHT	Phase II	[206–208] NCT04360174 NCT05335122
Latanoprost	PolyActiva Pty Ltd (Parkville, Australia)	﻿PA5108	Polytriazole hydrogel	Intracameral delivery; 6 months	OAG; OHT	Phase IIa	[209–211] NCT03604328 NCT04060758
Travoprost	Envisia Therapeutics (﻿Durham, NC, USA)	ENV515	Polymers with PRINT^®^ technology	Intracameral delivery; ND	OAG; OHT	Phase II	[212, 213] NCT02371746
CNTF	Neurotech Pharmaceuticals, Inc (Cumberland, RI, USA)	NT-501	Polymer membrane (ECT platform)	Intravitreal delivery; ND	Glaucoma	Phase II	[214–216] NCT01408472 NCT04577300 NCT02862938

PLGA, poly (lactic-co-glycolic acid); OAG, open-angle glaucoma; OHT, ocular hypertension; FDA, Food and Drug Administration; ND, not determined; CNTF, ciliary neurotrophic factor; ﻿ECT, encapsulated cell technology

## Challenges in clinical translation

The DDSs waiting to enter the market should possess treatment efficacy and be ideally be at least equal to or preferably better than conventional hypotensive eye drops [9]. One of the major reasons for the poor implementation of nano/micro pharmaceutical bench-to-bedside translation is the inadequate understanding of intraocular bio-performance. Unlike periocular or extraocular DDSs, once an intraocular DDS has been administrated into the eyes, it cannot be easily removed by any non-invasive method. Researchers may focus on pharmacological responses afore the biological impact of the materials administrated. The general physicochemical properties of nano/micro systems, such as particle size, shape, and surface charge, are usually under the focus of studies. However, the influence of the different intraocular environments, such as the history of intraocular surgery, repeated implantation, or vitreous liquefaction in elderly eyes on the various characteristics of DDSs has been rarely investigated [25, 103]. For instance, particle movement and clearance occur after administration in vitrectomized eyes [219] and aphakic eyes [105]. Even with Durysta™, a regulatory authority-approved intraocular DDS, ﻿large-scale clinical trials are still ongoing. The safety and efficacy of repeated administration of this implant have not been established. At least for now, the absence or rupture of the posterior lens capsule remains a contraindication to Durysta™ and retreatment is not allowed [141].

On the other hand, animal studies may not imply the same results in humans due to the significant difference between model eyes and human eyes [8]. The most common glaucoma models used in preclinical studies are rats and rabbits [220, 221], but both of them possess no macula [222]. Rats have a much smaller eyeball (approximately 6 mm in diameter) with a proportionally larger lens and corneal surface [222–225]. Larger animal models, such as rabbits, pigs, and dogs, have more similar eye size to human eyes, which allows the exploration of surgical techniques to administer DDSs, but anatomical and physiological differences remain remarkable [222, 226–233] and the costs of maintenance and facilities limit the use of large animals [222]. Rabbit have slender corneas, with fewer blinks and fewer tears [223, 234, 235]. They also lack PGA receptors and therefore the results of PGA-related studies based on rabbit models should be interpreted with caution [9]. Non-human primates are the nearest match to humans no matter their anatomy or physiology, but extremely high financial costs and ethical concerns are hindering their utilization [25, 222, 236]. More research on the in vivo fate of the DDS throughout its lifecycle (i.e. from initial implantation to complete degradation and retreatment) in actual human eyes, is required [25, 103, 225, 237].

Moreover, sufficient reproducibility and large-scale manufacturing techniques with minimal batch-to-batch variations need to be addressed before entering the market [8, 225]. The fabrication of DDSs often requires multiple steps involving different components and such a process can be time-consuming and expensive [8]. Maintaining repeatability via a robust process in a cost-effective manner is a challenge that requires multidisciplinary efforts.

Furthermore, the ways to deliver intraocular DDSs are invasive and they have increased risk of severe complications such as retinal detachment or entophthalmia. Even with the existing guidelines for intravitreal [238–242] and intracameral [243] injection, standard operating procedures (SOP) and procedure guidelines for each different DDS need to be established and should be easy to conduct in clinical practice. ﻿Kompella et al. suggested that one of the reasons why intravitreal injection can affect the field of ophthalmology is the establishment of its procedure guidelines and the compatible innovative design of its injectors [9, 238–242]. This point may also work for the translation of DDSs because all of these guidelines and developed devices improve the overall safety and patient acceptance of the treatment [9]. Moreover, how the DDSs will be administrated is also important. The ease of administration and maximized consideration of patient comfort contribute to adequate patient adherence [9].

## Future perspective

### ﻿Personalized drug delivery system

Considering that glaucoma is a multifactorial chronic retinal neurodegeneration, the combination of therapeutic substances targeting different pathophysiological mechanisms in glaucomatous pathology may be more effective compared with monotherapy [9, 22, 157]. Co-delivery of multiple drug agents that possess different physicochemical properties is difficult with conventional solvents (e.g. normal saline solution and glucose solution), but it is feasible with nanocarriers. For instance, Chan et al. developed a thermosensitive PLGA-PEG-PLGA copolymer to deliver hydrophobic and hydrophilic compounds (rhodamine B and coumarin 6) simultaneously. The drug concentration was at a high level for up to 4 weeks after a single sub-conjunctival injection [244]. In the future, personalized multi-drug therapy tailored to the physiological profile of each patient based on nano/micro drug carriers may become a routine choice of treatment.

### Nano-in-micro system (hybrid drug delivery system)

Nano-in-micro (NIM) system refers to the formation of a hybrid system by embedding or entrapping NPs into micro-matrixes such as hydrogels, microspheres or micelles [8]. Compared with a single-originated nano/micro system, a hybrid drug delivery system retains the advantages of its components, while minimizing its respective disadvantages. In addition, the entrapped NPs enlarge the total surface area for attracting drug agents [8]. For instance, when NPs possess relatively poor biocompatibility are incorporated with polymers with high biocompatibility, outer polymer matrixes may protect the embedded NPs and drug cargo in living tissues, consequently ameliorating the drug release profile and reducing the biotoxicity [8]. Another example is MSN, where the payload is easy to diffuse out of the porous channels before reaching the targeted sites from bare particles due to the open porous structure [165]. To protect the drug cargo from early release, Lyu et al. incorporated bevacizumab (BEV)-loaded MSNs into cyclosporine A-loaded PLGA-PEG-PLGA thermogel matrix [164]. In vitro BEV release study showed a burst release of BEV (about 77%) from BEV-loaded MSNs during the first 48 h, while only 33% of BEV was released from BEV-loaded MSNs embedding in thermogel during this period.

### Smart stimuli-responsive system

The term “smart” refers to the ability of DDS to provide a controlled release of the drug cargo at the exact time and site required in response to stimuli [158]. The stimuli can be exogenous (e.g. temperature gradient, light, magnetic field, ultrasound, electric field), or endogenous (e.g. pH change, enzyme activity) [8, 158]. ﻿Smart stimuli-responsive delivery systems can provide precise site-specific delivery in a controllable manner with minimal side effects or toxicity, which remains challenging for conventional NPs [8]. In addition, programmed sequential release and multi-responsiveness can also be achieved when combining different stimuli-responsive components with NIM strategies [8, 158]. Versatile smart stimuli-responsive DDSs have been well-developed for various diseases (for reviews, refer to [8, 158, 245]), but with few studies on glaucoma.

## Concluding remarks

Glaucoma is a sight-threatening disease affecting the all-age population worldwide. The major obstacles to glaucoma treatment with topical eye drops include the non-adherence of patients and limited bioavailability of medications, especially for a chronic disease that requires life-long treatment every single day. Nanomaterial-based drug delivery strategies hold great promise because they are powerful in achieving sustained release, target delivery, improved bioavailability, reduced side effects, and enhanced treatment efficacy. Despite promising prospects and expectations of intraocular drug delivery systems, there remain problems to be addressed, such as reliable and cost-effective scale-up production, safety and efficacy studies throughout their lifecycle in different intraocular environments, before regulatory authority approval and commercialization. To complete the successful bench-to-bedside translation, further extensive investigations are still required to answer the above-mentioned questions. With significant multidisciplinary research efforts, clinicians and patients can look forward to additional therapeutic options that may be available in the coming years.

## Data Availability

Not applicable.
